# Sensitive Detection and Quantification of Oxygenated Compounds in Complex Samples Using GC-Combustion-MS

**DOI:** 10.1021/acs.analchem.4c01858

**Published:** 2024-06-19

**Authors:** Javier García-Bellido, Montserrat Redondo-Velasco, Laura Freije-Carrelo, Gaëtan Burnens, Mariella Moldovan, Brice Bouyssiere, Pierre Giusti, Jorge Ruiz Encinar

**Affiliations:** † Department of Physical and Analytical Chemistry, University of Oviedo, 33006 Oviedo, Spain; ‡ TotalEnergies One Tech Belgium, Zone Industrielle C, 7181 Feluy, Belgium; § International Joint Laboratory−iC2MC: Complex Matrices Molecular Characterization, TRTG, 76700 Harfleur, France; ∥ Universite de Pau et des Pay de l’Adour, E2S UPPA CNRS, IPREM, Institut des Sciences Analytiques et de Physico-chimie pour l’Environnement et les Matériaux UMR5254, 64053 Pau, France; ⊥ TotalEnergies, TotalEnergies Research & Technology Gonfreville, 76700 Harfleur, France

## Abstract

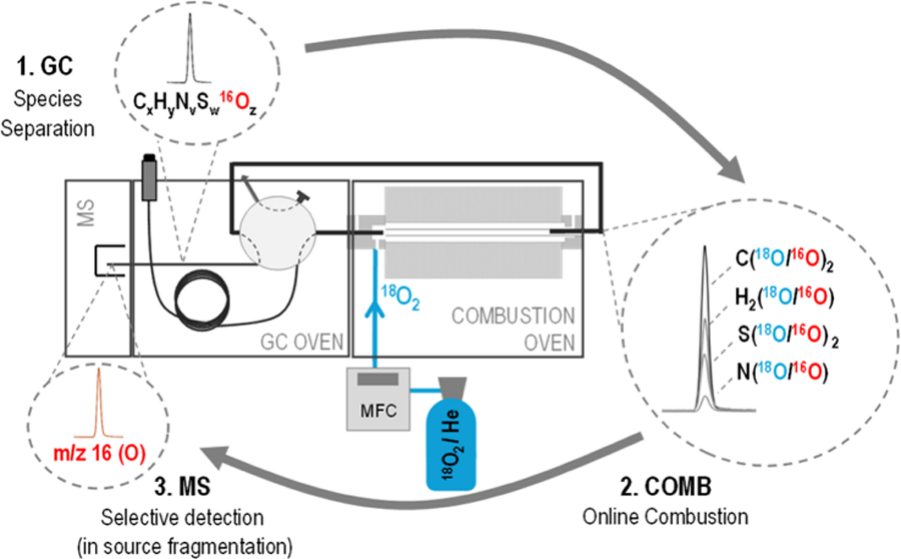

This work introduces a new element-selective gas chromatography detector for the accurate quantification of traces of volatile oxygen-containing compounds in complex samples without the need for specific standards. The key to this approach is the use of oxygen highly enriched in ^18^O as the oxidizing gas in a combustion unit (800 °C) that allows us to directly and unambiguously detect the natural oxygen present in the GC-separated compounds through its incorporation into the volatile species formed after their combustion and their subsequent degradation to ^16^O in the ion source. The unspecific signal due to the low ^16^O abundance in the oxidizing gas could be compensated by measuring the *m*/*z* 12 that comes as well from the CO_2_ degradation. Equimolarity was proved with several O-containing compounds with different sizes and functionalities. A detection limit of 28 pg of injected O was achieved, which is the lowest ever reported for any GC detector, which barely worsened to 55 and 214 pg of O when the oxygenate partially or completely coeluted with a very abundant matrix compound. Validation was attained by the analysis of a SRM to obtain accurate (99–103%) and precise (1–4% RSD) results. Robustness was tested after spiking a hydrotreated diesel with 10 O-compounds at the ppm level, which could be discriminated from the matrix crowd and quantified (mean recovery of 102 ± 9%) with a single generic standard. Finally, it was also successfully applied to easily spot and quantify the 33 oxygenates naturally present in a complex wood bio-oil sample.

## Introduction

Oxygen is one of the most commonly occurring constituents of organic compounds, and its determination is nowadays crucial in a wide variety of scientific disciplines (e.g., petroleomics, metabolomics, clinical, environmental sciences) and various industrial applications (e.g., hydrocarbon processing, new energies, natural gas and biogas, and pharmaceutical, chemical, and additive manufacturing).^1^ In fact, not only the total oxygen present in the sample is required but also the characterization of the different oxygen-containing compounds^2^ and their individual concentrations is harder. In particular, the determination of the individual amounts of the different oxygen-containing compounds present in new feedstocks and sustainable biofuels is of paramount importance nowadays to assess their potential uses and optimize the upgrading hydrotreatment required to remove the amount of oxygen present.^3^ The reason lies in the high reactivity of the various oxygen functional groups; for example, carboxylic acids can be corrosive, while aldehydes and ketones can lead to the formation of gums.^4^ Such detailed quantitative characterization together with a deep understanding of the combustion process will be critical to select better fuel candidates and develop more efficient catalytic production routes.^5^ Another consideration is the importance of oxygen-containing compounds in clinical samples. Current gas chromatography–mass spectrometry (GC-MS)-based metabolomics approaches could actually benefit enormously from the selective detection and quantification of oxygen metabolites in target biological samples, such as breath or body fluids.^6^

Gas chromatography (GC) is the gold standard technique to separate a huge number of volatile constituents in complex samples. Unfortunately, chromatographic peaks corresponding to oxygenated compounds frequently overlap with those from the matrix constituents, hampering their detection and quantification. One conventional solution^4^ to this problem is resorting to selective preconcentration or extraction methodologies to enrich oxygenates in purified fractions prior to the GC analysis [ASTM D4815]. Similarly, recent work has shown that comprehensive two-dimensional GC is very efficient for the separation of most of the oxygenates in complex samples.^7,8^ While these methods are certainly powerful, they involve long analysis times and sample manipulation and still require adequate detectors for identification and accurate quantification. In this context, another complementary approach to measuring the concentrations of oxygenated compounds in complex samples is to resort to selective GC detectors that respond only to O-containing compounds. Four main types of oxygen-selective GC detectors have been described so far. The oxygen-flame ionization detector (O-FID) converts oxygenated compounds in a catalytic reactor, first into carbon monoxide and then into methane, which is then detectable by a conventional FID.^9^ The absolute detection limit should be lower than 1 ng of O s^–1^ and selectivity and linearity are adequate (>10^6^ and 10^3^, respectively) [ASTM D5599–22]. However, it is restricted to simple samples since it is seriously affected by coelutions with other matrix compounds. Equimolar response is not warranted as relative response factors for every family of compounds must be computed in advance as in a regular FID. The Fourier transform infrared (FTIR) detector measures the absorption of IR energy by specific functional groups on the GC analyte.^10^ For oxygenated compounds, the C-0 stretching region is monitored [ASTM D5986–96]. Unfortunately, selectivity is low (<1000), presumably owing to broad overlapping gas-phase IR absorption bands and the detection limit achievable (100 ng) restricts its application fields. The atomic emission detector (AED) has many of the characteristics of an ideal oxygen-selective detector. Microwave-induced plasmas atomize the compounds eluting from the GC column and excite their constituent atoms producing characteristic emissions. Unfortunately, capability of multielement characterization is lost when oxygen is monitored since it demands for extremely high-purity He because minute quantities of air and water produce a background emission signal that can severely decrease oxygen selectivity and sensitivity.^11^ The detection limit is adequate (1 ng O);^12^ however, it suffers from significant matrix (carbon-char) and quenching effects when analyzing complex unresolved samples. Equimolarity is not good either since deviations as high as 40% are possible.^13^ Finally, it is clear that mass spectrometry is a potent technique for analyzing mixtures, especially when using high-resolution instruments. Unfortunately, its identifying capability is limited in complex samples for the screening of O-containing compounds due to chromatographic coelutions and isobaric interferences.^11^ Moreover, ionization is compound-dependent, making necessary the use of specific standards to perform the quantification of every individual O-containing compound.^14^ Therefore, in spite of the pressing need for the accurate determination of the oxygen-containing compounds present at trace levels in complex samples targeted in many scientific and industrial fields, there is still no widely accepted method for this difficult challenge.

In this work, we have developed a highly sensitive instrumental approach to detect selectively and quantify O-containing compounds in complex samples without the need to resort to specific standards. Our approach makes use of isotopically enriched ^18^O_2_ during the combustion step that takes place between the GC separation and the ionization in the MS instrument. The proof-of-concept strategy to stand out the O-containing compounds from the organic crowd and perform their accurate quantification is demonstrated. Excellent agreement with the certified values of a soy-based biodiesel (SRM 2772) with the use of simple generic internal standards demonstrates its quantitative accuracy and precision. Moreover, we successfully applied this approach to detect and quantify 10 different oxygenated compounds previously spiked at the low (7–50)-ppm range to a complex diesel and those naturally present in a pine wood bio-oil.

## Experimental Section

### Reagents, Solutions, and Materials

Dodecane (C12; 99%), tetradecane (C14; 99%), nonadecane (C19;98.5%), eneicosane (C20; 99.8%), butylbenzene (BB; 99%), butanol (C4OL;100%), pentyl butyrate (PB; 99.3%), acenaphthene (AC; 98.5%), cyclohexanone (Cy6ONE; 99%), 2-ethoxyethyl acetate (EtO; 99%), hexylbutyrate (HB; 97%), 1-heptanol (C7OL; 98%), 1-octanol (C8OL; 99%), acetophenone (A; 99.8%), benzaldehyde (B; 99%), dimethylmaleate (DiMAL; 98.7%), benzothiophene (BT; 97%), methylbenzothiophene (MBT, 96%), phenethylacetate (PhA; 97%), dibutylaniline (DBA; 99%), dibutylsulfide (DS; 98%), dibenzofurane (DBF; 100%), dimethylphthalate (DPh; 99.5%), indole (I, 99%), 1-methylindole (1MI, 97%), 3-methylindole (3MI, 98%), and dibenzothiophene (DBT, 98%) were purchased from Merck. The SRM 2772 soy-based B100 biodiesel (NIST) was used to validate the methodology. Helium (purity 99.999%) was purchased from Air Liquide. Two different gas mixtures were used as the combustion gas, ^16^O_2_/He (0.3%, v/v) from Linde and ^18^O_2/_He (1% v/v, 97% ^18^O_2_ enriched) from Westfalen AG. Real samples were provided by TotalEnergies Raffinage Chimie, a hydrotreated diesel sample, and aliquots from effluents were taken at different times along the hydrotreatment of a wood bio-oil.

### Instrumentation

For GC separations, experimental conditions are summarized in Table S1.

GC-combustion-MS instrument: A Shimadzu GC-combustion-MS instrument, based on a GC-MS QP-2020NX instrument, as described in Figure S1, was used. The instrument was configured with a split/splitless inlet and an electron ionization source operated at 70 eV. The modification consisted of a combustion oven that allows the complete combustion of the analytes by using an alumina tube (400 mm length × 3 mm width × 0.5 mm ID; Elemental Microanalysis) with Pt wires as catalyzers. The installation of an automatic 6-way valve allows the system to work as a standard GC-MS system. An additional He makeup flow (ca. 1.7 mL min^–1^) was introduced to protect the capillary interface and reduce peak broadening.

## Results and Discussion

**Figure 1:**
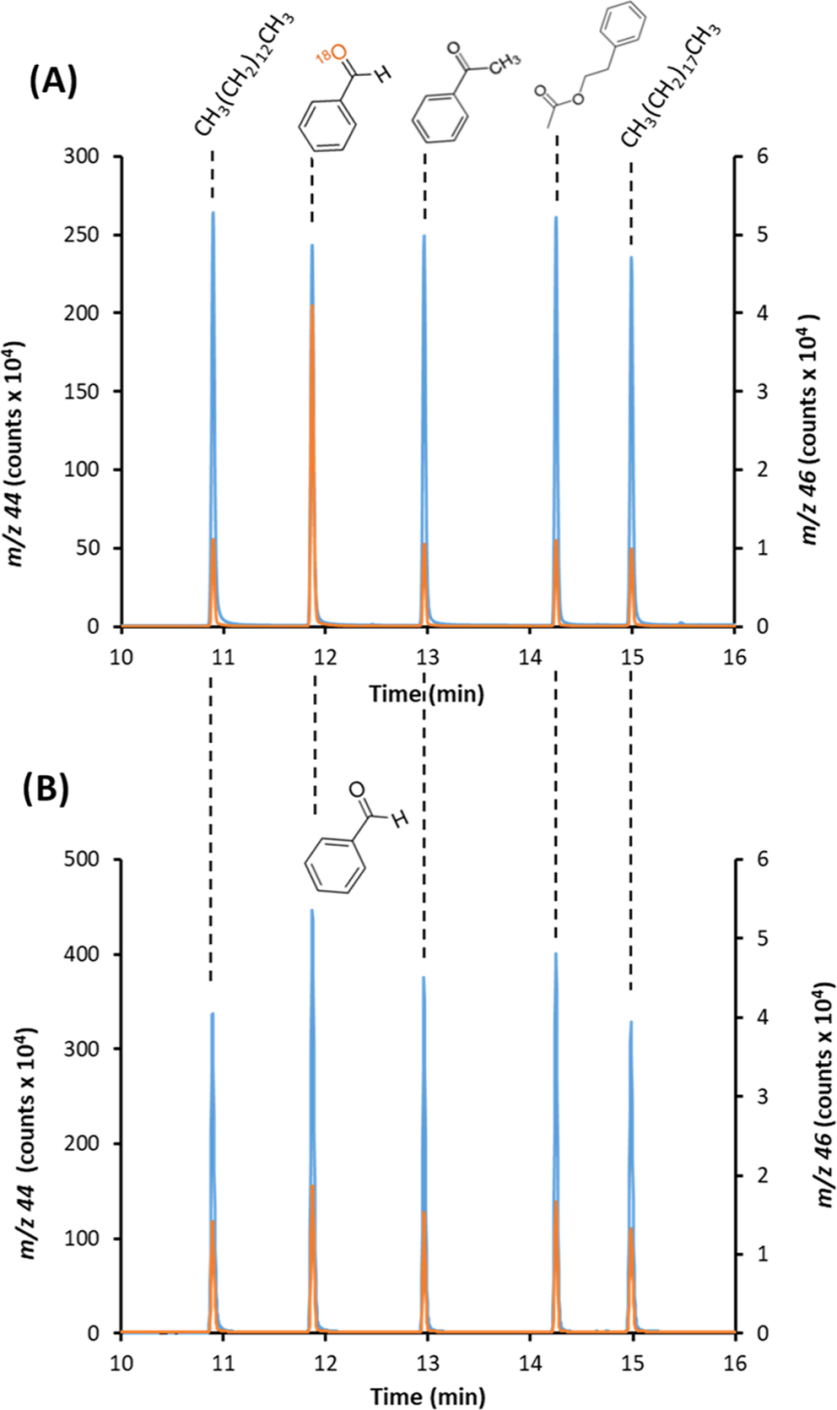
GC-combustion-MS chromatogram obtained with natural oxygen as combustion gas for (A) mixture of benzaldehyde containing isotopically enriched oxygen (Ab^18^O = 97.1%) with two alkanes (tetradecane and nonadecane) and two compounds containing natural oxygen, acetophenone and phenethyl acetate. (B) Same mixture but substituting the isotopic benzaldehyde with one containing natural oxygen. Orange and blue profiles correspond to 46 and 44, respectively.

The guiding principle is the GC detector introduced in 2009,^15^ which is able to provide generic universal quantification of organic compounds, while maintaining the inherent structural elucidation capabilities of MS by simply actuating a switching valve. The combustion interface developed and installed in a regular GC–MS instrument allowed for the online quantitative conversion of each and every organic compound eluting from the column into CO_2_ before the ionization.^16^ The system was considerably improved over time based on the idea that other volatile species, such as H_2_O, SO_*x*_, and NO_*x*_ (if S and N are present), would be produced as well in the combustion oven together with CO_2_, opening the gate to parallel H-, S-, and N-selective detection.^17^Figure S1 shows the detailed schematics of the system. Therefore, oxygen detection became the one stumbling block to the long-wished GC detector combining structural identification (MS) with compound-independent calibration, both universal (C, H) and element-selective (N, S, and O). As it has been already pointed out before, oxygen detection is already a very challenging task and, to make matters worse, our strategy uses an on-line flow of 0.4 mL min^–1^ oxygen diluted in He (0.3% v/v) to produce the combustion of the organic compounds previously separated in the GC column. In order to avoid peak broadening, such combustion is on-line produced inside a narrow ceramic tube containing two Pt wires (catalyst) and heated at ≥800 °C. The resulting volatile combustion species are then brought to a manually actuated high-temperature six-way valve, installed inside the GC, which in turn allows directing them to the ion source of the MS instrument. Such a valve allows also bypassing the combustion furnace when necessary, which enables the setup to work under GC–MS (Figure S1A) or GC-combustion-MS (Figure S1B) configurations.^18^ The expected products for a complete oxidation of organic compounds^19^ with O_2_ as the oxidant would then comprise
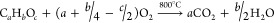
1Of course, other N- and S-containing volatile species would be formed at high temperatures (i.e., NO_*x*_ or SO_*x*_) if present in the compound. Therefore, in principle, it should be expected that the O present in the original organic compounds would be distributed between the oxidized volatile species formed together with the O used in the combustion (in excess). This scenario makes oxygen detection impossible unless the O used in the combustion and the target O present in the compounds are different. In order to check this starting hypothesis, we resorted to one compound isotopically enriched in ^18^O (Ab^18^O = 97.1%), benzaldehyde. For comparison purposes, we mixed it with two alkanes (C14 and C19) and two compounds containing natural oxygen (Ab^16^O = 99.76% and Ab^18^O = 0.205%, see Table S2), acetophenone and phenethyl acetate. The mixture was then injected into the GC-combustion-MS system described above. The resulting chromatogram is shown in Figure 1A. As can be clearly observed, the intensity profiles at *m*/*z* 44 (corresponding exclusively to ^12^C^16^O_2_) and *m*/*z* 46 (corresponding mostly to ^12^C^16^O^18^O, assuming that Ab^17^O is negligible in both the natural and enriched oxygens) differed significantly in benzaldehyde, where the intensity for *m*/*z* 46 was much higher in comparison to the other four compounds (please note that the signal profile at *m*/*z* 48 was too low for being properly measured). The 44:46 ratios measured (*n* = 5) for the alkanes (241 ± 2 and 241 ± 4, 2 SD), where oxygen had been incorporated exclusively from the combustion gas, and those for the natural O-containing acetophenone and phenethyl acetate (239 ± 4 and 242 ± 4, 2 SD) matched perfectly the theoretical natural 44/46 ratio computed using the natural oxygen and carbon abundances (243 ± 1). In contrast, the 44:46 ratio decreased to 63 ± 1 (2 SD) in the case of the benzaldehyde due to the incorporation of the enriched ^18^O originally present in the CO_2_ molecules formed after combustion, greatly increasing the signal at *m*/*z* 46 as clearly shown in Figure 1A. Notably, the chromatogram obtained for the same mixture containing benzaldehyde with natural oxygen instead led to the same 44 and 46 profiles for all the compounds (Figure 1B), the 44:46 ratio obtained for natural benzaldehyde being 247 ± 4 (2 SD) in this case.

Once demonstrated that isotopically labeled oxygen compounds can be detected when using natural oxygen as the combustion gas, we decided to reverse the reasoning and explore the use of isotopically labeled O_2_ (Ab^18^O = 97%) as the combustion gas to detect compounds that contain natural oxygen. Initially, a mixture of 12 O-compounds (including alcohols, aldehydes, esters, ethers, and carbonyls with saturated and aromatic structures), 2 alkanes, and 1 aromatic compound was prepared in hexane and injected in triplicate. As shown in Figure S2 and detailed in Table S3, the noncontaining O-compounds produced CO_2_ molecules where the abundance of the *m*/*z* 46 (^12^C^16^O^18^O) was rather low because of the low isotopic abundance of ^16^O in the isotopically enriched (^18^O_2_) combustion gas (however, still higher than the nominal 3% due to air contamination in the system). Instead, the O-containing compounds produced CO_2_ molecules with higher 46:48 and 44:48 ratios due to the significant contribution of the natural ^16^O originally present in the compounds. Unfortunately, neither the 44 or 46 peak areas nor the 44:46 and 44:48 ratios followed any clear relationship with the O concentration present in each species. Traditional equations of isotope dilution are also difficult to use in this case as they apply when the amount of the isotopically enriched (natural or radioactive) tracer (^18^O_2_ in our case) is accurately known and controlled and an equilibrium is established between the natural element and the known amounts of the isotopic element added within the blend that is analyzed.^20^ In fact, the isotopically labeled species (^18^O_2_ diluted in He) is used here^21^ as a reaction reagent, added on line and in huge excess before the combustion furnace, to achieve the complete combustion of the organic compounds of the sample eluting from the GC. Therefore, the mass flow of the sample typically computed in on-line isotope dilution applications, where the enriched spike is not reacting with the sample and is simply added as a quantification standard,^22−24^ cannot be computed here. Both the natural oxygen originally present in the organic compounds of the sample and the isotopic oxygen used are distributed in the volatile species formed (CO_2_ and H_2_O). In fact, the amount of isotopic-^18^O incorporated into the volatile species formed after combustion for each eluting compound depends on the amount of natural oxygen originally present and their corresponding elemental composition. This is because the higher the number of C and H in the compound, the higher the amount of ^18^O incorporated into the CO_2_ and H_2_O molecules formed. It is for all of these reasons that oxygen detection in the O-compounds is possible only after comparing the signals obtained for the noncontaining O-compounds used as internal standards.

**Figure 2:**
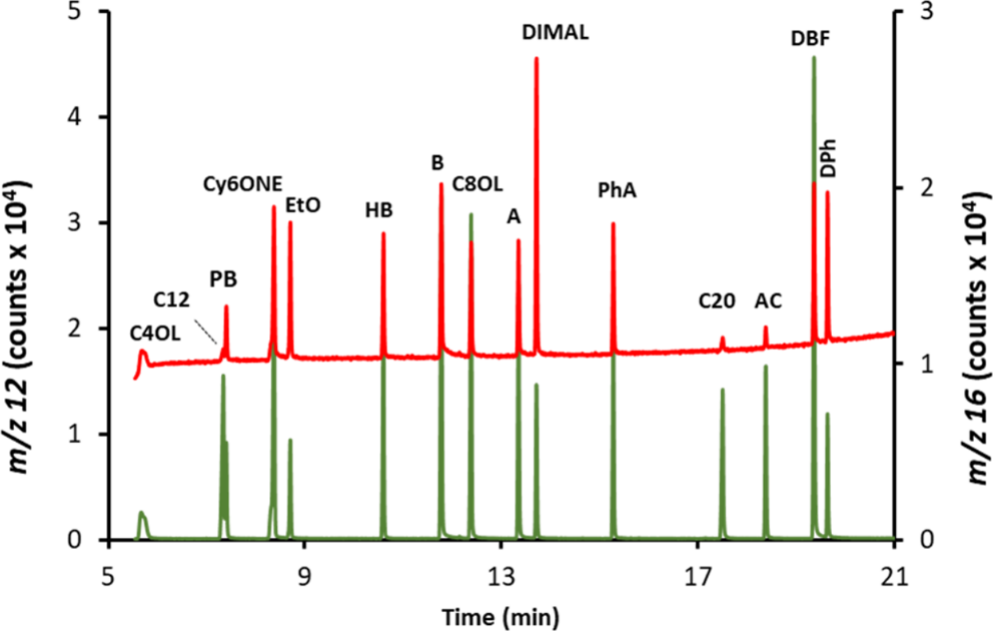
GC-combustion-MS chromatogram at *m*/*z* 16 and 12 (red and green, respectively) obtained for a mixture of three noncontaining (C12, C20, and AC) and 12 O-containing (C4OL, PB, Cy6ONE, EtO, HB, B, C8OL, A, DiMAL, PhA, DBF, DPh) compounds using ^18^O-enriched oxygen (1% in He) as the combustion gas. Compounds’ abbreviations and compound concentrations are given in the Experimental Section. Oxygen concentration ranged from 7.1 to 34.2 μg O g^–1^ with an average value of 17 μg O g^–1^.

Interestingly, we observed a signal at *m*/*z* 16 that seemed to be related to the presence of natural oxygen (Ab^16^O = 99.8%) in the compound and could be directly used for quantification purposes. The origin of such an analytical signal might be the in-source fragmentation of CO_2_ and H_2_O to O, generally established and shown in the corresponding NIST reference spectra with abundances close to 10 and 0.9%, respectively. This is clearly observed in Figure 2, which shows the profile at *m*/*z* 16 of the chromatogram obtained for the mixture under study. Notably, it is apparent from Figure S3A that peak areas at *m*/*z* 16 already followed a quite linear trend (*R*^2^ = 0.968) with the O concentration for each compound. As expected, the *m*/*z* 16 peaks obtained for the two alkanes and the aromatic are significantly lower but still significant because of the low abundance of ^16^O (3%) in the enriched combustion gas, which could be further increased by small air leaks within the instrumental system. This unspecific contribution is responsible for the intercept of the *m*/*z* 16 calibration (Figure S3A) and is ruled by the size of every compound since the higher the number of C, the higher the number of CO_2_ molecules produced and the higher the amount of residual ^16^O incorporated. Notably, the in-source fragmentation also brings about the production of a C signal at *m*/*z* 12 (shown in the NIST reference spectrum with an abundance of 8.7%) that reflects compound size and could be used as an internal standard to estimate such an unspecific contribution. The profile at *m*/*z* 12 is also given in Figure 2. As expected, every compound, containing oxygen or not, provides a signal at *m*/*z* 12, depending exclusively on its C concentration. In fact, as shown in Figure S3B, there is good linearity (*R*^2^ = 0.989) between the peak areas for all of the compounds present in the mixture and their corresponding *C* concentration.

**Figure 3:**
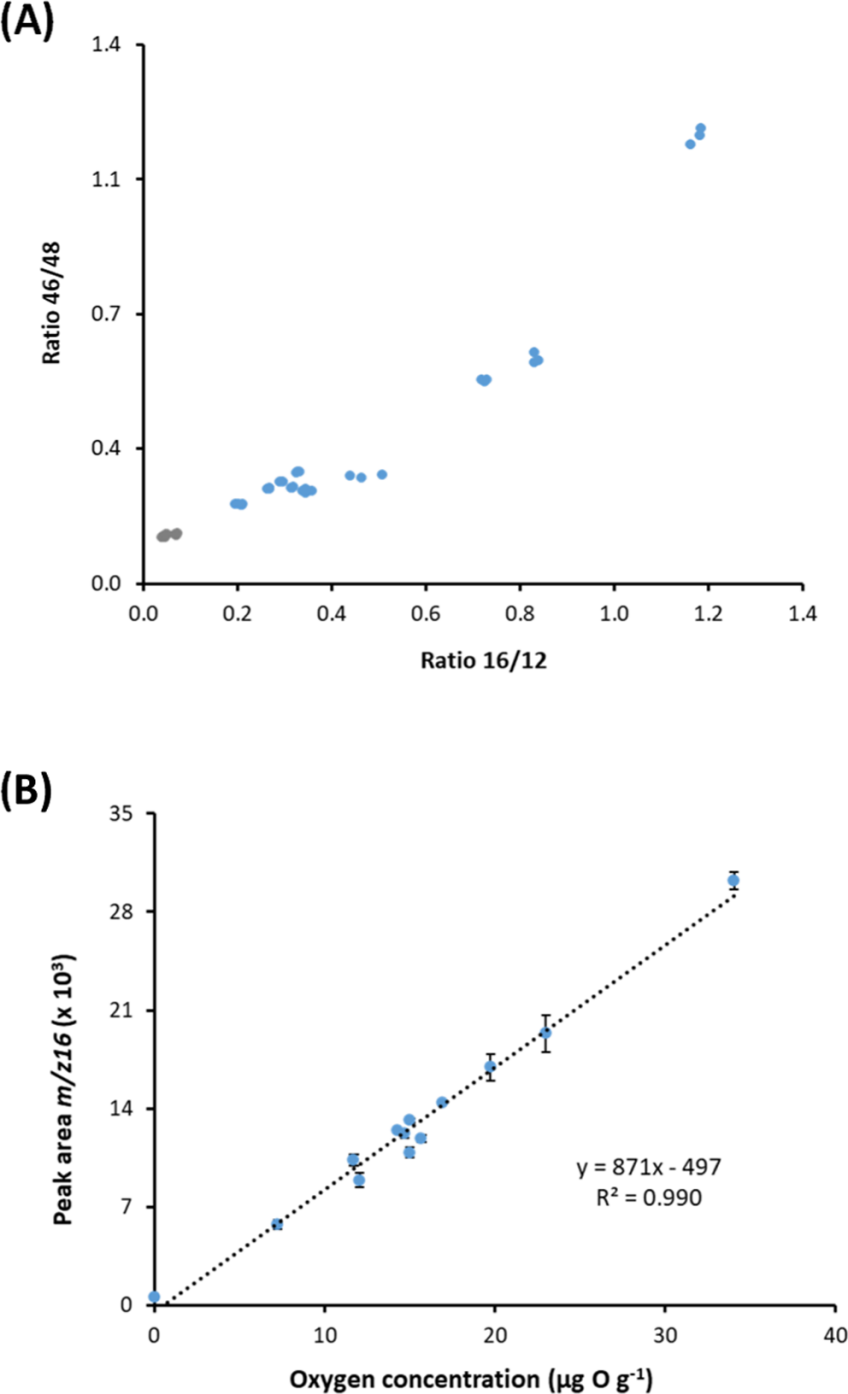
(A) Plot of the intensity ratios 16:12 vs 46:48 measured for each chromatographic peak shown in Figure 2 showing three noncontaining (gray circles: C12, C20, and AC) and 12 O-containing compounds (blue circles: C4OL, PB, Cy6ONE, EtO, HB, B, C8OL, A, DiMAL, PhA, DBF, DPh). The results for the three replicates are plotted together (*n* = 45). (B) Calibration curve of the net peak areas at *m*/*z* 16 obtained for the same mixture using the quantification strategy developed. Uncertainty bars correspond to 1 SD (*n* = 3).

In the search for an analytical strategy to selectively screen for O-compounds in mixtures, we decided to plot the discriminating peak intensity ratios (easily provided by software of the instrument) 16:12 vs 46:48 (*m*/*z* 44 intensity was too low to be measured properly for lower oxygen concentrations). Figure 3A, where the results for the three replicates are plotted (*n* = 45), demonstrates that noncontaining O-compounds (gray circles) are well discriminated and gathered in close formation in the lower left corner of the graphic. In contrast, the O-compounds (blue circles) are classified along the two-axis depending on their O to C ratio. To the best of our knowledge, this is the first time that molar ratios O/C are measured in separated GC peaks through their corresponding elemental signals (*m*/*z* 12 and 16).

Before starting to develop the generic quantitative strategy, we needed to ensure that the enrichment of the oxidizing gas used for combustion was constant along the gradient. Initially, a set of experiments were conducted using a mixture containing 11 alkanes from C12 to C20. The chromatogram is shown in Figure S4. Peak area ratios at 46, 47, and 48 obtained for each alkane were used to compute for the corresponding oxygen abundances (considering carbon natural isotope abundances). It is apparent from Table S4A that ^16^O-abundance was slightly but continuously increasing along the gradient from 6.21% (C12) to 6.64% (C20), while the ^18^O-enrichment decreased from 92.60 to 92.17%, likely due to a tiny change in the residual air leaks in the system along gradient. Interestingly, we observed that the 32:34 ratio (measured simultaneously at their corresponding *m*/*z*), that could be somehow related to the ^16^O/^18^O ratio, was also slightly increasing along the gradient. In fact, as can be seen in Table S4B, after application of the 32:34 trend to correct for the slight increase in the ^16^O-abundance, both the ^18^O and ^16^O abundances measured for the alkanes remained completely stable (from 92.60 to 92.61% and from 6.21 to 6.20%, respectively). Therefore, we decided to apply this correction in our quantitative strategy, as the peak areas measured at *m*/*z* 16 should also follow the same trend. Interestingly, such an alkane mixture could be used as well to assess if protonation of CO_2_ takes place in the ion source due to the water cogenerated,^25^ that could impact the sensitivity of our approach. In fact, the experimental ^49^CO_2_/^48^CO_2_*m*/*z* ratio measured could be used as a proxy of the natural ^13^C/^12^C ratio. We found that the 49:48 *m*/*z* ratio obtained for the mixture of the alkanes (0.0107 ± 0.0002, *n* = 9) was equivalent to the representative natural ^13^C/^12^C ratio (0.0108 ± 0.0008, IUPAC). Of course, this could occur only if protonation of the very abundant *m*/*z* 48 is negligible.

The next step was to correct our analytical signal at *m*/*z* 16 for the unspecific contribution of the low ^16^O abundance in the ^18^O-enriched combustion gas. For that purpose, the mean 16:12 peak area ratio measured in the noncontaining O-compounds present in the mixture and taken as internal standards of C (IS-C) was used to estimate the unspecific contribution at 16 for each O-compound (oxy) when multiplied by their corresponding signals (peak areas) at 12
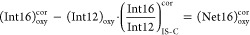
2where the superscript “cor” refers to the peak area at 16 previously corrected using the 32:34 trend as explained above and “Net16” corresponds to the ultimate analyte signal that comes exclusively from the natural oxygen (Ab^16^O = 99.8%) originally present in the target compound. We could then plot the (Net16)_oxy_^cor^ for each O-compound present in the mixture against their corresponding O concentration, and the resulting calibration graph is given in Figure 3B. As can be seen, the developed quantitative strategy provides a clear advance on the linearity achievable that now rises up to *R*^2^ = 0.990. Another striking benefit to emerge from Figure 3B is that the approach proposed seems to be fully species-independent (ca. equimolar) since 11 very different O-compounds in size, functional groups, and aromaticity provide a very similar response factor (i.e., calibration slope). This feature opens the door to compound-independent calibration, especially interesting in complex samples with lots of unknown O-compounds.

In order to explore further the selectivity of the approach proposed, we created another set of mixtures containing additionally *N* (dibutilaniline, indol, 1- and 3-methyl indol)- and S-containing (benzothiophene, dibenzothiophene, methylbenzothiophene, and dibutylsulfide) compounds. We also included another alkane (nonadecane), aromatic (butylbenzene), and O-containing compound (1-heptanol). These new mixtures were also analyzed in triplicate. In total, 200 chromatographic peaks were processed along different working days (5), including 93 of noncontaining and 107 of O-containing compounds (with average and lowest concentrations of 14 and 4.1 ppm of O, respectively). As can be seen in Figure S5, the 16:12 vs 46:48 global plot allowed us to distinguish clearly the O-compounds. In fact, after considering all the noncontaining compounds, we could estimate the mean and standard deviation for each ratio. Then, according to the criteria of the 99% confidence interval (means +2.33σ, *n* = 93), any compound providing ratios above the corresponding limits (red dotted lines in Figure S5) was classified as an O-compound. Interestingly, as can be clearly seen in the inset of the figure, N-compounds provided high 16:12 values (but still lower than the values of the O-compounds), likely due to the formation of nitrogen hydride species during electron ionization (as observed in the NIST mass spectra of NO). Surprisingly, although analyses were carried out on different working days and the dispersion of the data is higher, the 16:12 ratio still allowed complete discrimination (0% false positives or negatives). However, under such stringent conditions, the 46:48 ratio failed to classify one triplicate of 1-butanol as an O-compound, likely due to its poor peak shape (see Figure 2) and classified one triplicate of dibutylaniline just in the borderline. This result, comprising a large population of compounds measured on different days, further strengthened our confidence in the selectivity of the approach proposed.

The detection limit was then calculated as the ultimate limit of our strategy to discriminate between O- and noncontaining compounds. We are aware that O-containing compounds can be present in complex real samples at much lower concentrations than other matrix compounds, so we wanted to explore as well how such a detection limit is influenced by coelution. For that purpose, a low concentrated (ca. 0.5–0.6 μg O g^–1^) solution of an oxygenate (2-pentyl butyrate) was spiked with a closely eluting noncontaining O-compound (dodecane) at increasing carbon concentration ratios (1.1, 5.6, 9.3, 14.7, and 18.4). As the concentration ratio increases, peak resolution worsened (in both GC-MS and GC-combustion-MS chromatograms) from almost complete resolution (0.9) at equal concentrations (Figure S6A) to a tiny shoulder (Figure S6B–D) that finally disappeared at the higher concentration ratio (Figure S6E). Then, the three times standard deviation of the unspecific ratio  computed for a later eluting alkane (C20) used as a reference was translated into O concentration using the difference of the corresponding ratios  and the O concentration of the O-compound. As can be seen in Table S5, the detection limit computed this way turned out to be as low as 28 pg of O injected when chromatographic resolution allowed fully individual integration of the coeluting peaks (Figure S6A). It got worse when integration ended up being more critical as the oxygen peak became a shoulder, ranging from 53–58 pg of O, and finally rose to 214 pg of O when coeluting peaks had to be integrated together (Figure S6E). To the best of our knowledge, even during complete coelution, these are the lowest detection limits for O ever published for a GC detector. It is worth noting that there is still room for improvement if we raise the isotopic enrichment of the oxygen used as the combustion gas and reduce further the already low air leaks in the system. Of course, as long as there is any shoulder or peak distortion that could indicate the presence of two different peaks, individual MS spectra (insets to right panels in Figure S6) can be taken and detection of the trace of the O-containing compound could be attained by conventional GC-MS. However, as clearly seen in Figure S6E, when the coeluting alkane was in almost 20 times excess with regards to the oxygenate, there was no way of telling the presence of two peaks, and therefore, only one single overall MS spectrum was taken where no trace of the oxygenate could be detected. Finally, we wanted to explore as well the accuracy of the quantification of such O-containing compounds when coeluting. To assess this issue in detail, we spiked our complete coeluting mixture of 2-pentyl butyrate and dodecane at a C molar ratio of 18.4 (Figure S6E) with a generic O-compound (dimethyl phthalate) to carry out the quantification. Quantitative results, expressed as recoveries, were strikingly good (93 ± 5%, 1 SD, *n* = 5) despite the low concentration of the target O-compound (0.56 ppm of O) and the great excess of the interfering peak.

For validation purposes, we resorted to a standard reference material (NIST, SRM 2772) consisting of a soy-based B100 nonfossil biodiesel with certified and reference values for several fatty acid methyl esters (FAMEs). After adequate dilutions with hexane, the SRM sample was spiked with nonadecane and dimethyl phthalate as generic internal standards of C (IS-C) and O (IS-O), respectively, and analyzed in quintuplicate. Figure S7A shows a representative GC-combustion-MS chromatogram obtained at masses of 16 and 12. The five main FAMEs, C16:0, C18:0, C18:1, C18:2, and C18:3, were detected. Unfortunately, position isomers C18:1(*n*-9) and C18:1(*n*-7) could not be chromatographically resolved so they were quantified together. As expected, the 16:12 vs 46:48 plot allowed us to distinguish clearly the O-compounds, both the FAMEs and IS-O, from the IS-C (Figure S7B). The oxygen concentration determined for each FAME was translated into compound concentration for comparison purposes to the SRM values. Table 1 shows the excellent agreement between the concentrations found and the certified values for every quantified FAME with recoveries ranging from 99 to 103%. In addition, the precision ranged from 0.9 to 4.3% RSD, depending on the concentration level. Such results validate our approach and demonstrate its potential for the accurate and precise quantification of O-containing compounds using simple generic standards.

**Table 1:** Quantitative Recoveries Obtained for the FAME Determination in SRM 2772 Using Nonadecane and Dimethylphthalate as Generic Internal Standards of C and O^a^

	SRM 2772	GC-Combustion-MS	
FAME compound	certified, mg g^–1^	found, mg g^–1^	recovery, %
methyl palmitate (C16:0)	107 ± 2	110 ± 2	103
methyl stearate (C18:0)	43.0 ± 2.7	43.9 ± 3.8	102
methyl oleate (C18:1, *n*-9)	233 ± 6	249 ± 10^c^	101^c^
methyl vaccinate (C18:1, *n*-7)	14.3 ± 1.5		
methyl linoleate (C18:2, *n*-6)	523 ± 17	521 ± 7	100
methyl linolenate (C18:3, *n*-3)	69.3 ± 2.6^b^	68.6 ± 3.3	99

a Concentrations are referred to the original SRM sample. Uncertainty corresponds to the 95% confidence interval (*n* = 5).

b Reference value.

c Sum of C18:1(*n*-9) and C18:1(*n*-7).

**Figure 4:**
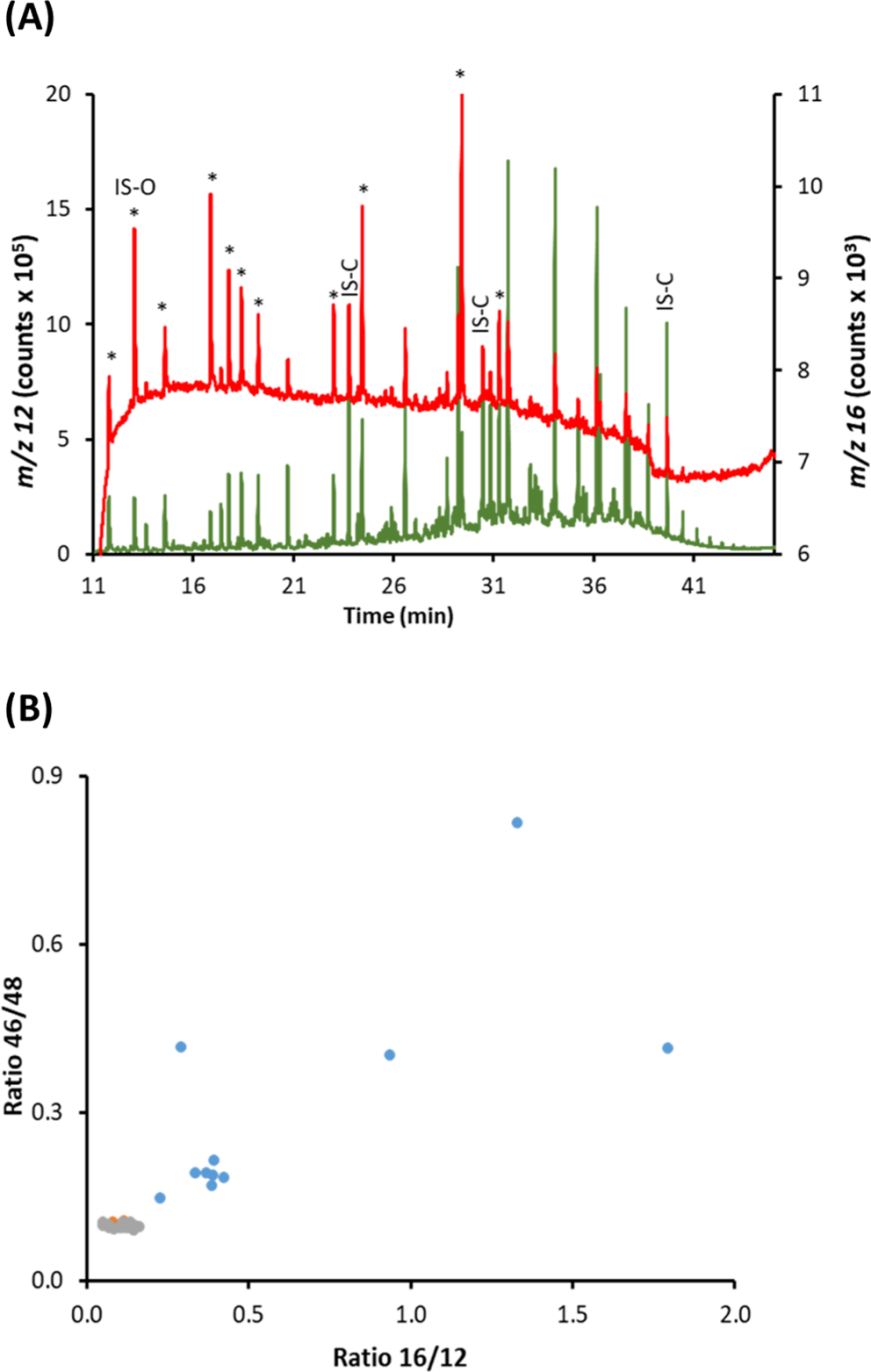
(A) GC-combustion-MS chromatogram of the hydrotreated diesel spiked with 10 O-compounds (labeled with an asterisk). 2-Ethoxyethyl acetate (EtO) was used as an nternal standard (IS-O). (B) Plot of the ratios 16:12 vs 46:48 measured for each chromatographic peak detected in the spiked diesel sample. Color code: IS-C added (orange), C-matrix peaks detected (gray), and spiked O-compounds (blue).

The applicability of the proposed approach to real sample analysis was further validated with a diesel sample that was previously hydrotreated to completely remove the heteroatoms. Such a diesel sample was spiked with 10 O-compounds at the μg·g^–1^ level. In parallel, 2-ethoxyethyl acetate (EtO) was spiked as well to be used as IS-O. In addition, three noncontaining O-compounds (C12, C20, and AC) were spiked to serve as IS-C. Figure 4A shows the GC-combustion-MS chromatogram obtained at *m*/*z* 16 and 12 (that matches pretty well with the universal GC–MS profile). Up to 35 significant peaks were detected at *m*/*z* 16. It is interesting to note that only those matrix compounds with concentrations higher than ca. 5 μg C g^–1^ in the injected sample produced a significant unspecific signal at *m*/*z* 16. After application of the plot 16:12 vs 46:48 as the discrimination strategy (Figure 4B), the spiked 11 O-containing compounds were unambiguously classified as O-containing compounds (labeled with an asterisk in Figure 4A) and clearly distinguished from the three noncontaining O-compounds spiked and the other 21 components of the matrix producing an unspecific signal at *m*/*z* 16. Quantitative results are given in Table 2. In spite of the sample complexity, recoveries obtained were adequate, ranging from 82 to 112% with a mean recovery value of 102%. Although some of the spiked O-containing compounds coeluted with matrix C-containing peaks, especially the last three (phenethylacetate, dimethylphthalate, and dibenzofurane) eluting in the unresolved complex mixture (UCM), quantification was still accurate. This is because any unspecific contribution at *m*/*z* 16 coming from coeluting C-containing peaks is corrected after measuring the *m*/*z* 12 and applying the ratio  measured for the internal standard.

**Table 2:** Concentrations Found and Quantitative Recoveries Obtained for the 10 O-compounds Spiked to the Hydrotreated Diesel^a^

O-compound spiked	added, μg g^–1^	found, μg g^–1^	recovery, %
cyclohexanone	13.9	12.4	89
benzaldehyde	12.6	10.4	82
dimethylmaleate	36.7	37.5	102
pentyl butyrate	15.3	15.9	104
acetophenone	12.7	14.2	112
1-octanol	10.5	11.4	109
hexylbutyrate	12.9	13.2	103
phenethylacetate	25.2	26.9	107
dimethylphthalate	44.1	47.1	107
dibenzofurane	9.84	10.2	104

a 2-Ethoxyethyl acetate (EtO) was spiked as an internal quantification standard (IS-O). Compounds are given in the elution order (see Figure 4).

**Figure 5:**
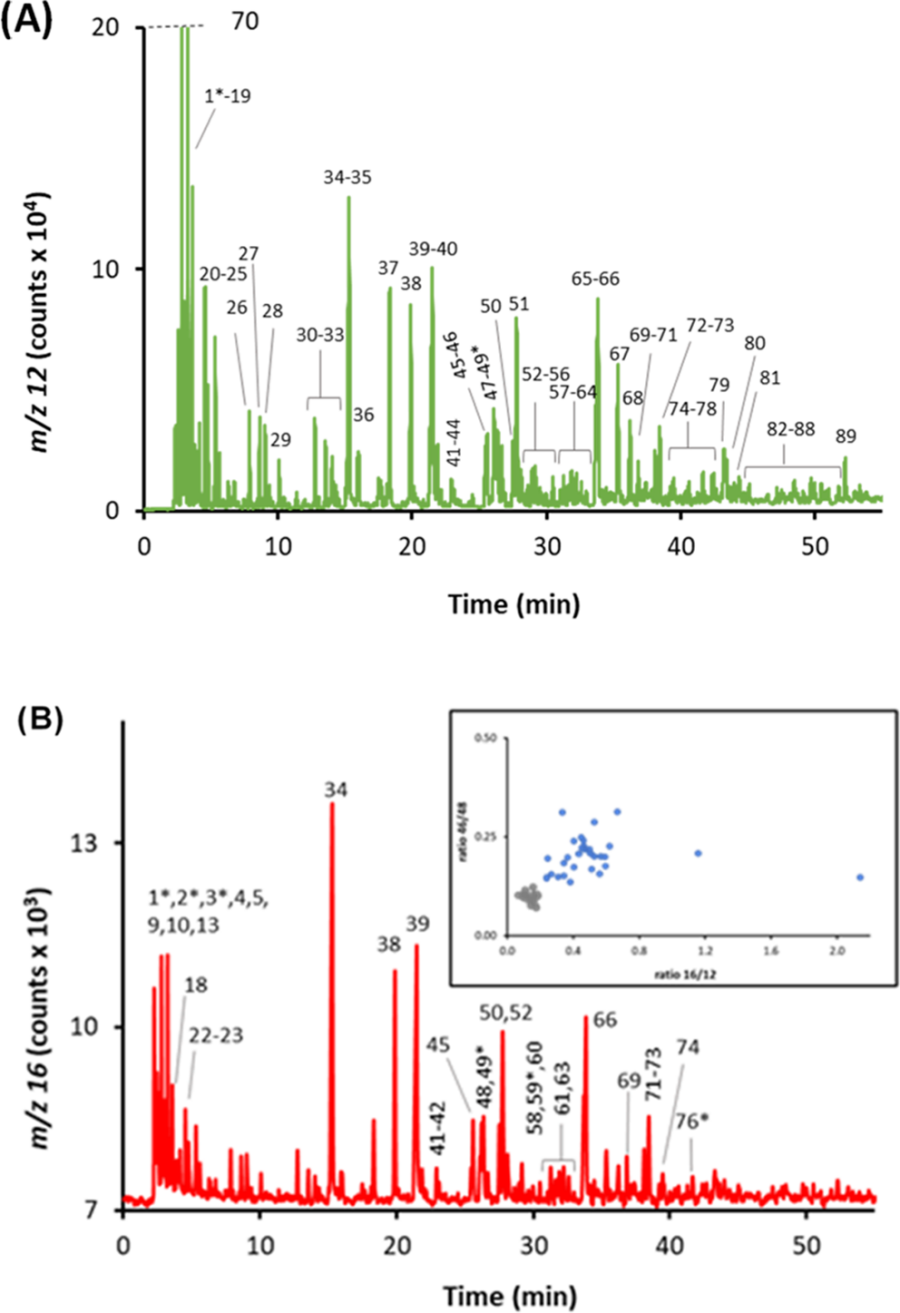
GC-combustion-MS chromatogram at *m*/*z* 12 (A) and *m*/*z* 16 (B) of the pine wood bio-oil. The 89 detected peaks are numbered in (A), while the 33 O-containing compounds selectively identified and quantified are numbered in (B). The inset corresponds to the plot of the ratios 16:12 vs 46:48 measured for each chromatographic peak detected, the gray and blue dots being the C-matrix peaks detected and the identified O-containing compounds (corroborated by GC-MS), respectively.

Finally, we wanted to demonstrate its applicability to the determination of oxygenates naturally present in complex real samples. For that purpose, we applied it to the understanding of the upgrading by hydrotreatment of a wood bio-oil^26^ (obtained from reactive catalytic fast pyrolysis of loblolly pine). Two hydrotreated effluents taken at different time points on the reactor stream (after 72.5 and 144.5 h) were analyzed. The objective was to assess the catalyst deactivation. Complexity of the sample is clearly shown in Figure S8 by the crowded chromatogram obtained by GC-MS, which is similar to the universal GC-combustion-MS profile obtained at *m*/*z* 48. In fact, up to 89 significant peaks could be detected using the *m*/*z* 12 profile (Figure 5A), 68 of whom produced a detectable signal as well at *m*/*z* 16 (Figure 5B). After application of the discrimination strategy (inset of Figure 5B), 33 and 35 peaks were unambiguously classified as O-containing and non-O-containing compounds, respectively. Notably, direct analysis by GC-MS only led to the identification of 27 O-containing peaks (MS similarity, NIST library). We then assessed the six peaks classified as O-containing peaks whose regular averaged EI-MS spectra did not provide any significant match to any oxygenate compound (within the 10 first options and with scores lower than 80%). We found out that when obtaining point-by-point MS spectra at particular sections (ends) of those controversial peaks, we could obtain positive identifications of oxygenates. The global list of the 33 O-containing and 62 non-O-compounds finally identified is given in Table S6, including the six O-compounds detected thanks to the discrimination power of the strategy proposed (labeled with an asterisk in Figure 5B). In this case, and due to the extreme complexity of the chromatograms shown in Figure 5, we decided to carry out the quantification of the 33 different oxygen-containing compounds detected in the bio-oil sample using an independent sample containing a mixture of two generic standards (1-octanol and dimethylphthalate). Overall oxygen contents (sum of individual O-compounds) obtained for the effluents taken at 72.5 and 144.5 h were 17.3 and 34.5 mg O g^–1^, respectively, which clearly suggest a loss in the hydrotreatment performance likely due to catalyst deactivation.^26^ Interestingly, such bulk results were about 30% lower than those obtained using an elemental analyzer (27.1 and 42.9 mg of O g^–1^, respectively), which makes sense taking into account the incomplete vaporization in the GC injector typically observed for complex bio-oil samples.

## Conclusions

In conclusion, a sensitive and robust strategy was developed to screen oxygen-containing compounds among matrix compounds and accurately quantify them without resorting to specific standards. The strategy involves the use of GC to separate the sample compounds, isotopically enriched ^18^O_2_ as the combustion gas, and final analysis by mass spectrometry. The usage of a distinct isotopic oxygen as the combustion gas allows the detection of the target natural oxygen originally present in the compounds. It is incorporated into the volatile species formed (e.g., CO_2_, H_2_O), which partially break down in the ion source, producing an analytical signal at *m*/*z* 16. Notably, the limitation due to the low unspecific *m*/*z* 16 observed for highly concentrated noncontaining O-compounds because of the residual abundance (ca. 6%) of ^16^O in the isotopic combustion gas used can be corrected by measuring the ^12^C signal (also coming from in-source degradation). The measurement of the 16:12 ratio in a noncontaining ^16^O compound, which is used as an IS, provides the tool to compute the unspecific contribution of such residual ^16^O in the O-compounds. In fact, the measurement of the 16:12 ratios in every detected GC peak turned out to be an easy to compute and perfect discriminating factor (0% of false positives and negatives) to accurately screen for O-compounds within the samples analyzed, including the bio-oil and diesel, despite the low oxygen concentrations (low-ppm range) assayed. The accuracy of the method, tested in standards, SRM and a spiked diesel sample, is comparable to that of other established element-selective detectors in GC, such as ECD, NCD, or SCD.^11^ Finally, its potential to detect and quantify O-containing compounds in complex unresolved samples was proved by monitoring the hydrotreatment process of a wood bio-oil. Last but not least, taking into account the price of the isotopic oxygen bottle and assuming an analysis time of 1 h and the working flow of 0.4 mL min^–1^, the approximate cost of analysis is 0.35 €. This low cost together with the saving in analytical standards (only generic O-containing and C-containing standards are necessary) results in a very cost-effective approach.

Applications can be foreseen in a wide variety of fields, ranging from the petroleum and chemical (polymer and plastic) industries to quantitative metabolomics, where the determination of the great and rising variety of O-containing compounds is increasingly important. This work is the last step in the development of an innovative multipurpose GC detection system (GC-comb-MS) featuring element-selective detection (C, H, N, S, and O) with generic quantification in complex samples, while maintaining the structural elucidation power of mass spectrometry.^17,18^ In fact, it can be regarded as the first approach that enables the online and simultaneous ultrasensitive elemental quantification of every individual volatizable (GC) organic compound present in complex samples. Of course, such tremendous potential to provide elemental fingerprints for individual compounds in complex samples will be boosted even further when combined with the huge separation power of multidimensional GC.

## Supporting Information Available

Experimental conditions, scheme of the GC-combustion-MS instrument, CO_2_ peak areas ratios and chromatograms for the mixture of O-containing compounds and alkanes, oxygen and carbon isotopic abundances, calibration curves and detection limit, and chromatograms and list of identified compounds for real samples (PDF)
