# Supplementary material for: Sensitive Detection and Quantification of Oxygenated Compounds in Complex Samples Using GC-Combustion-MS
Source: Anal Chem. 2024 Jun 19;96(26):10756–64. doi: 10.1021/acs.analchem.4c01858 (PMC11223096; doi:10.1021/acs.analchem.4c01858)
Supplement: Supplementary file 1 — ac4c01858_si_001.pdf [file ac4c01858_si_001.pdf]

Supporting Information

Sensitive detection and quantification of oxygenated compounds in complex samples using GC-combustion-MS

Javier García-Bellido,[a‡] Montserrat Redondo-Velasco,[a‡] Laura Freije-Carrelo,[b,c] Gaëtan Burnens,[b,c] Mariella Moldovan,[a] Brice Bouyssiere,[c,d] Pierre Giusti[c,e]* and Jorge Ruiz Encinar[a]*

[a] Department of Physical and Analytical Chemistry, University of Oviedo, 33006, Oviedo, Spain

[b] TotalEnergies One Tech Belgium, Zone Industrielle C, 7181 Feluy, Belgium

[c] International Joint Laboratory – iC2MC: Complex Matrices Molecular Characterization, TRTG, 76700 Harfleur, France

[d] Universite de Pau et des Pay de l'Adour, E2S UPPA CNRS, IPREM, Institut des Sciences Analytiques et de Physico-chimie pour l'Environnement et les Matériaux UMR5254, 64053 Pau, France

[e] TotalEnergies, TotalEnergies Research& Technology Gonfreville, 76700 Harfleur, France

* Jorge Ruiz Encinar: ruizjorge@uniovi.es and Pierre Giusti: pierre.giusti@totalenergies.com

‡ These authors contributed equally to this work.

Table of Contents

Table S1. Experimental conditions used for the quantitative analysis of O-containing compounds by GC-combustion-MS and GC. **S4**

Table S2. Natural isotope abundance of carbon and oxygen (IUPAC). The expanded uncertainties listed in parentheses include the range of probable isotope-abundance variations among different materials as well as measurement uncertainties. **S5**

Table S3. Oxygen concentration (µg O mL^{-1}) and 46 ($^{12}C^{16}O^{18}O$) to 48 ($^{12}C^{18}O_2$) peak area ratios for the mixture of twelve O-compounds (including alcohols, aldehydes, esters, ethers and carbonils with saturated and aromatic structures) with three noncontaining O compounds (two alkanes and one aromatic compound, highlighted in red) shown in Figure S3. Uncertainty corresponds to 1 SD (n=3). **S6**

Table S4. Oxygen isotope abundances computed for a mixture of eleven alkanes (Figure S5) based on the 46, 47 and 48 peak area ratios. Uncertainty corresponds to 1 SD (n=3), without (A) and with (B) correction using the 32/34 trend........ **S7**

Table S5. Relative (ng O g^{-1}) and absolute detection (pg O) limit for an oxygen-containing compound (2-Pentyl butyrate) obtained under conditions of partial and complete co-elution with a non-containing O-compound (dodecane) present at different concentration ratios (mixtures A-E). Corresponding chromatograms are shown in Figure S6. .... **S8**

Table S6. List of the compounds identified in the aliquot of the effluent taken from the hydrotreatment process of a wood bio-oil (144.5 h of the catalyst life). The numbering (#) corresponds to the peak numbers given in Figure 5. Oxygen-containing peaks are highlighted in red. Asterisks indicates the six oxygenates that co-eluted with a more abundant non-O containing compound that could only be discriminated after application of the screening approach using the GC-combustion-MS peak ratios (16/12 and 46/48). **S9-S10**

Figure S1. Scheme of the six-way valve and its connections within GC-combustion-MS. Position A (GC-MS mode): GC effluent is directly sent to the MS. Position B (GC-combustion-MS mode): GC effluent is first mixed online with the O_2/He combustion gas and the He makeup-flow before entering the combustion furnace and finally brought to the MS. **S11**

Figure S2. GC-combustion-MS chromatogram obtained for a mixture of three noncontaining (C12, C20 and AC) and twelve O-containing (C4OL, PB, Cy6ONE, EtO, HB, B, C8OL, A, DiMAL, PhA, DBF, DPh) compounds using ^{18}O-enriched oxygen (1% in He) as combustion gas. Compounds' abbreviations are given in the Experimental Section. Oxygen concentration ranged from 7.1 to 34.2 µg O g^{-1} with an average value of 17 µg O g^{-1}. Orange, blue, pink profiles correspond to 46, 44 and 48, respectively. **S12**

Figure S3. Oxygen (*m/z* 16, A) and Carbon (*m/z* 12, B) calibration curves obtained from the GC-combustion-MS chromatogram shown in Figure 2, which consists of a mixture of three noncontaining (C12, C20 and AC) and twelve O-containing (C4OL, PB, Cy6ONE, EtO, HB, B, C8OL, A, DiMAL, PhA, DBF, DPh) compounds using ^{18}O-enriched oxygen (1% in He) as combustion gas. Uncertainty bars correspond to 1 SD (n=3). Compounds' abbreviations and compound concentrations are given in the Experimental Section... **S13**

Figure S4. GC-combustion-MS chromatogram obtained for a mixture of eleven alkane compounds (C12-C20) using 18O-enriched oxygen (1% in He) as combustion gas. Blue, orange and pink profiles correspond to *m/z* 44, 46 and 48, respectively. Compounds' abbreviations and compound concentrations are given in the Experimental Section. Concentration of the compounds was 16 µg C g^{-1}. **S14**

Figure S5. Plot of the ratios 16/12 vs 46/48 measured for each chromatographic peak detected in several mixtures of noncontaining and O-containing compounds and analyzed in triplicate on different working days. In total, 200 chromatographic peaks were processed, including 93 noncontaining (3 alkenes, 1 aromatic, 4 N-compounds, 4 S-compounds) and 107 O-containing peaks (13 O-compounds). Red dotted lines correspond to the limits of the 99% confidence interval for the non-containing O-compounds. Oxygen concentrations ranged from 4 to 37 µg O g^{-1}, with an average value of 14 µg O g^{-1}. See experimental section for details. Color code: alkanes (grey), aromatics (yellow), N-compounds (purple), S-compounds (green) and O-compounds (blue). **S15**

Figure S6. GC-combustion-MS (left panels) and GC-MS (right panels) chromatograms corresponding to low concentrate solutions (ca. 0.5-0.6 µg O g^{-1}) of an O-containing compound (2-Pentyl butyrate, retention time: 11.73 min) spiked with a noncontaining O-compound (C12, retention time: 11.67 min) at increasing concentration ratios: 1.1 (A) , 5.6 (B), 9.3 (C), 14.7 (D) and 18.4 (E). Left panels: orange and dark blue traces correspond to signals at *m/z* 12 and 16, respectively in the GC-combustion-MS chromatogram. Right panels: pale blue corresponds to the TIC signal in the GC-MS chromatograms and insets correspond to the corresponding MS spectra at the retention times of the individual peaks (A-D) and the unique-global peak (E)........ **S17-S17**

Figure S7. A) GC-combustion-MS chromatogram of an aliquot of the Biodiesel SRM 2772 previously spiked with Nonadecane and Dimethylphtalate as generic Internal Standards of C (IS-C) and O (IS-O). B) Plot of the ratios 16/12 vs 46/48 measured for each chromatographic peak detected in the quintuplicate analysis. Color code: IS-C (grey), IS-O (blue) and FAMEs (orange, O-compounds)... **S18**

Figure S8. GC-MS (A) and GC-combustion-MS at m/z 48 (B) chromatograms of an aliquot of the effluent taken from the hydrotreatment process of a wood bio-oil (144.5 h of the catalyst life). **S19**

Table S1. Experimental conditions used for the quantitative analysis of O-containing compounds by GC-combustion-MS and GC.

Inlet temperature	250 ºC
Injection mode	SRM: Split 1:5 (Diluted 1:1350 in hexane)<br>Diesel: Splitless (Diluted 1:100 in hexane)<br>Bio-oil: Split 1:200
Injection volume	1 µL
Columns	Standards mixtures and SRM: SH-FameWax (30 m x 0.32 mm x 0.25 µm)<br>Diesel and bio-oil: SH1-MS (30 m x 0.25 mm x 0.25 µm)
Carrier gas	He (1.5 mL/min)
GC Oven temperature	SRM: 50 ºC (1 min) - 15 ºC/min to 250 (5min)<br>Diesel: 30°C (5 min) to 180°C at 5°C/min, 180°C to 320°C (10 min) at 15°C/min<br>Bio-oil: 50°C (5 min) to 320°C (10min) at 2°C/min
Acquisition mode	SIM: *m/z* 12, 16, 32, 34, 44-49
Combustion oven temperature	800 ºC
O_2/He flow	0.4 mL/min

Table S2. Natural isotope abundance of carbon and oxygen (IUPAC). The expanded uncertainties listed in parentheses include the range of probable isotope-abundance variations among different materials as well as measurement uncertainties.

Element	Mass number	Representative isotopic composition
C	12	0.9893 (8)
	13	0.0107 (8)
O	16	0.99757 (16)
	17	0.00038 (1)
	18	0.00205 (14)

Table S3. Oxygen concentration (μg O mL^{-1}) and 46 ($^{12}C^{16}O^{18}O$) to 48 ($^{12}C^{18}O_2$) peak area ratios for the mixture of twelve O-compounds (including alcohols, aldehydes, esters, ethers and carbonils with saturated and aromatic structures) with three noncontaining O compounds (two alkanes and one aromatic compound, highlighted in red) shown in Figure S2. Uncertainty corresponds to 1 SD (n=3).

Compound	O concentration (μg O mL^{-1})	Peak area ratio, 46/48
1-butanol	12.1	0.2787 ± 0.0042
Dodecane	0.0	0.1201 ± 0.0016
Pentyl butyrate	7.24	0.2385 ± 0.0026
Cyclohexanone	23.0	0.2891 ± 0.0006
2-Ethoxyethyl acetate	16.9	0.584 ± 0.015
Hexylbutyrate	15.7	0.2487 ± 0.0017
Benzaldehyde	19.8	0.2644 ± 0.0007
1-octanol	15.0	0.2051 ± 0.0012
Acetophenone	11.7	0.2459 ± 0.0011
Dimethyl maleate	34.1	1.163 ± 0.022
PhenetylAcetate	14.7	0.2415 ± 0.0026
Icosane	0.0	0.1265 ± 0.0018
Acenapthene	0.0	0.1276 ± 0.0026
Dibenzofurane	14.3	0.2059 ± 0.0008
Dimethylphtalate	15.0	0.5269 ± 0.0029

Table S4. Oxygen isotope abundances computed for a mixture of eleven alkanes (Figure S4) based on the 46, 47 and 48 peak area ratios. Uncertainty corresponds to 1 SD (n=3), without (A) and with (B) correction using the 32/34 trend.

A)

Compound	Without any correction		
	Ab ^{16}O	Ab ^{17}O	Ab ^{18}O
C12	6.21 ± 0.02	1.19 ± 0.01	92.60 ± 0.01
C13	6.27 ± 0.04	1.20 ± 0.001	92.54 ± 0.04
C14	6.31 ± 0.05	1.20± 0.006	92.49 ± 0.05
C15	6.33 ± 0.05	1.20± 0.003	92.48 ± 0.05
C16	6.40 ± 0.04	1.19± 0.004	92.41 ± 0.04
C17	6.45 ± 0.06	1.20± 0.005	92.35 ± 0.05
C18	6.51 ± 0.04	1.19± 0.004	92.30 ± 0.04
C19	6.56 ± 0.06	1.19± 0.004	92.25 ± 0.05
C20	6.64 ± 0.03	1.19± 0.007	92.17 ± 0.03
mean	6.41	1.19	92.40
SD	0.14	0.005	0.1
RSD (%)	2	0.4	0.2

B)

Compound	After correction using the 32/34 trend		
	Ab ^{16}O	Ab ^{17}O	Ab ^{18}O
C12	6.21 ± 0.01	1.19 ± 0.01	92.60 ± 0.003
C13	6.24 ± 0.02	1.20 ± 0.001	92.56 ± 0.02
C14	6.26 ± 0.03	1.20 ± 0.006	92.54 ± 0.04
C15	6.24 ± 0.04	1.20 ± 0.003	92.56 ± 0.03
C16	6.27 ± 0.03	1.19 ± 0.004	92.54 ± 0.02
C17	6.26 ± 0.04	1.20 ± 0.005	92.54 ± 0.04
C18	6.25 ± 0.02	1.19 ± 0.004	92.56 ± 0.02
C19	6.22 ± 0.04	1.19 ± 0.004	92.59 ± 0.04
C20	6.20 ± 0.02	1.19 ± 0.007	92.61 ± 0.02
mean	6.24	1.19	92.57
SD	0.02	0.005	0.03
RSD (%)	0.4	0.4	0.03

Table S5. Relative (ng O g^{-1}) and absolute detection (pg O) limit for an oxygen-containing compound (2-Pentyl butyrate) obtained under conditions of partial and complete co-elution with a non-containing O-compound (dodecane) present at different concentration ratios (mixtures A-E). Corresponding chromatograms are shown in Figure S6.

Mixture	Alkane to oxygenate ratio of carbon mass	DL computed: ng O/g (pg O)
A	1.1	43 (28)
B	5.6	83 (54)
C	9.3	89 (58)
D	14.7	82 (53)
E	18.4	327 (214)

Table S6. List of the compounds identified in the aliquot of the effluent taken from the hydrotreatment process of a wood bio-oil (144.5 h of the catalyst life). The numbering (#) corresponds to the peak numbers given in Figure 5. Oxygen-containing peaks are highlighted in red. Asterisks indicates the six oxygenates that co-eluted with a more abundant non-O containing compound that could only be discriminated after application of the screening approach using the GC-combustion-MS peak ratios (16/12 and 46/48).

#	Compound	RT (min)	#	Compound	RT (min)
1*	Formic acid	2.2	25	Cyclohexene,1-methyl	5.6
	Propane		26	Cyclohexene, ethyl	7.9
2*	Propane,2-nitro	2.3	27	Cyclohexene, 1-ethyl-	8.6
	Butane		28	p-xylene	9.0
3*	Acetone	2.4	29	o-xylene	10.1
	Isobutane		30	Cyclohexane, propyl-	12.8
4	1-Butanol	2.5	31	Benzene, propyl-	13.6
5	1-Propanol	2.7	32	Benzene, ethyl-methyl- isomer	14.1
6	Cyclopentane	2.8	33	Benzene, ethyl-methyl- isomer	14.2
7	Butane,2-3-dimethyl-	2.8	34	Phenol	15.4
8	Pentane,3-methyl	2.9	35	1H-Indene, octahydro-, cis	15.9
9	Furan,2-methyl-	3.0	36	Benzene,1,2,3-trimethyl-	16.1
10	2-Ethyl-oxelate	3.0	37	Indane	18.4
11	Cyclopentane, methyl-isomer	3.2	38	Phenol,2-methyl-	19.9
12	Cyclopentane, methyl-isomer	3.3	39	Phenol,3-methyl-	21.6
13	4-Hexen-1-ol, (Z)-	3.5	40	Indan,1-methyl	21.8
14	Cyclohexane	3.6	41	Phenol, 2,6-dimethyl-	23.0
15	Cyclohexene	3.8	42	Benzofuran, 2-methyl-	23.1
16	Cyclopentane,1,3-dimethyl-cis-	3.9	43	Dodecane	24.5
17	2-Undecane,3-methyl-, (Z)-	3.9	44	1H-Indene,2,3-dihydro-5-methyl-	25.5
18	Furan,2-ethyl-	4.0	45	Phenol, 2-ethyl	25.6
19	Heptane	4.2	46	Benzene, 1-methyl-2-(2-propenyl)-	25.8
20	Cyclohexane, methyl-	4.5	47	3,9-Dodecadiene	26.1
21	Cyclopentane, ethyl-	4.8	48	Phenol,3,5-dimethyl	26.2
22	cis-Hept-4-enol	4.8	49*	1H-Indene-1,2-diol,2,3-dihydro-cis-	26.4
23	5,10-Dioxatricyclo [7.10.0(4,6)] decane	5.3		Naphthalene, 1,2,3,4-tetrahydro-	
24	Toluene	5.3	50	Phenol, 4-ethyl-	27.5
51	Naphthalene	27.8	71	Phenol,2-(1-methylpropyl)-	38.1

52	Phenol, 3-ethyl-	28.2
53	1H-Indene, dihydro-dimethyl-isomer	29.0
54	1H-Indene, dihydro-dimethyl-isomer	29.2
55	2-ethyl-2,3-dihydro-1H-indene	29.5
56	1H-indene,2,3-dihydro-1,6-dimethyl-	30.0
57	Naphthalene, 1,2,3,4-tetrahydro-2-methyl-	30.5
58	Phenol,2-propyl	31.3
59*	Phenol,2,4,6-trimethyl-<br>2-ethyl-2,3-dihydro-1H-indene	31.6
60	Phenol, -ethyl-methyl- isomer	31.9
61	Phenol, -ethyl-methyl- isomer	32.3
62	Naphthalene, 1,2,3,4-tetrahydro-1,5-dimethyl-	32.5
63	Phenol, -ethyl-methyl- isomer	32.6
64	1H-indene,2,3-dihydro-4,7-dimethyl-	33.0
65	Naphthalene, 1,2,3,4-tetrahydro-5-methyl-	33.8
66	Phenol,3-propyl	33.9
67	Naphthalene, methyl-isomer	35.4
68	Naphthalene, methyl-isomer	36.3
69	Benzaldehyde, 2-ethyl-	36.9
70	Naphthalene, 1,2,3,4-tetrahydro-2,7-dimethyl-	37.2

72	1H-Inden-5-ol,2,3-dihydro-	38.5
73	Phenol, 2-(1-methylpropyl)-	38.6
74	Phenol, 3,5-diethyl-	39.5
75	Naphthalene, 2-ethenyl-	40.7
76*	5,8,11-Eicosatriynoic acid, metyl ester<br>Naphthalene, 1-ethyl-	41.7
77	Naphtalene, dimethyl-isomer	42.4
78	Naphtalene, dimethyl-isomer	42.5
79	Naphtalene, dimethyl-isomer	43.3
80	Naphtalene, dimethyl-isomer	43.5
81	Naphtalene, dimethyl-isomer	44.4
82	Naphtalene, dimethyl-isomer	45.2
83	Naphthalene, 1,4,6-trimethyl-	48.5
84	Naphthalene, 1,6,7-trimethyl-	49.7
85	Naphthalene, trimethyl-isomer	50.0
86	Heneicosane	50.6
87	Azulene, 4,6,5-trimethyl-	51.1
88	Naphthalene, trimethyl-isomer	51.8
89	Fluorene	52.3

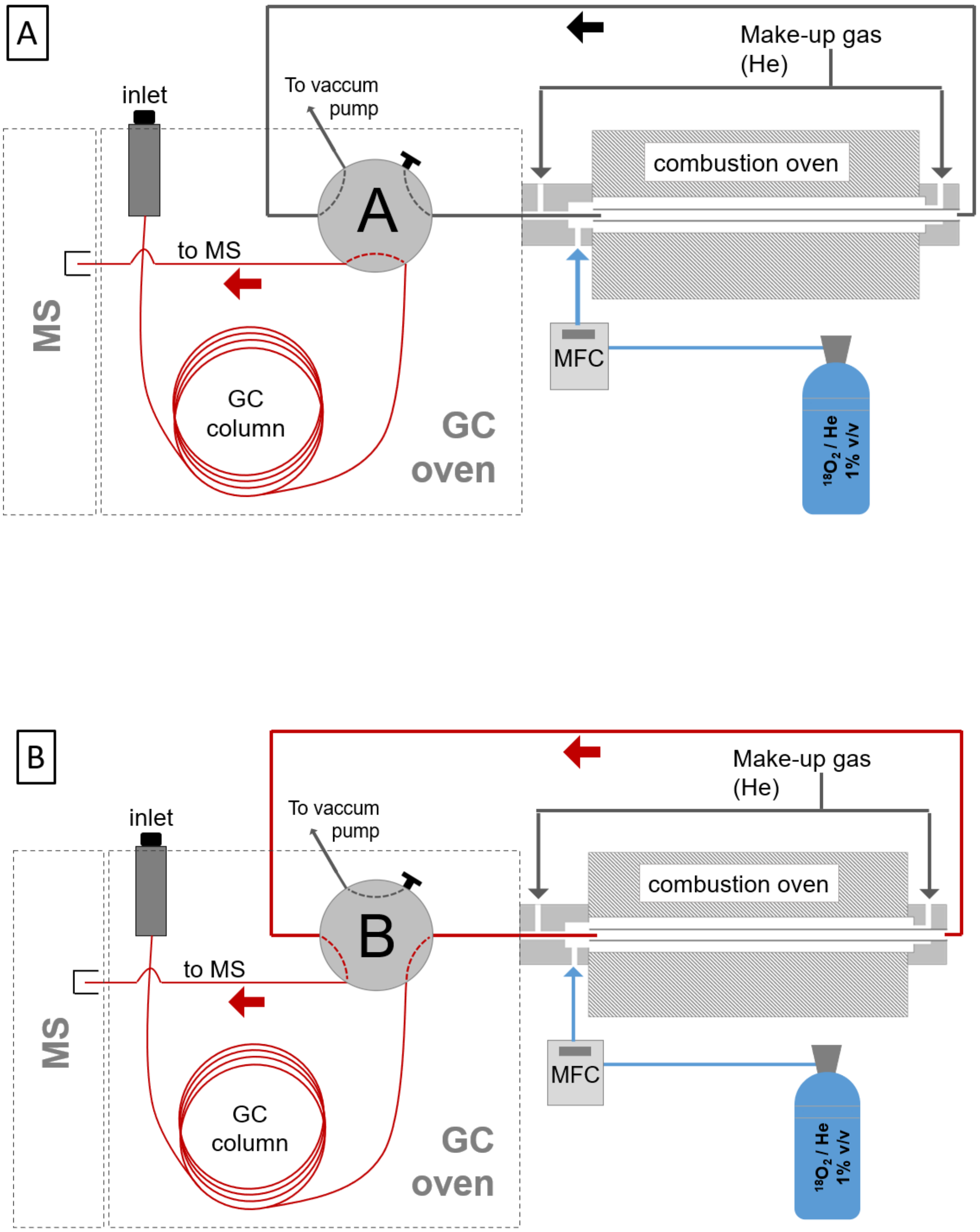

Figure S1. Scheme of the six-way valve and its connections within GC-combustion-MS. Position A (GC-MS mode): GC effluent is directly sent to the MS. Position B (GC-combustion-MS mode): GC effluent is first mixed online with the O_2/He combustion gas and the He makeup-flow before entering the combustion furnace and finally brought to the MS.

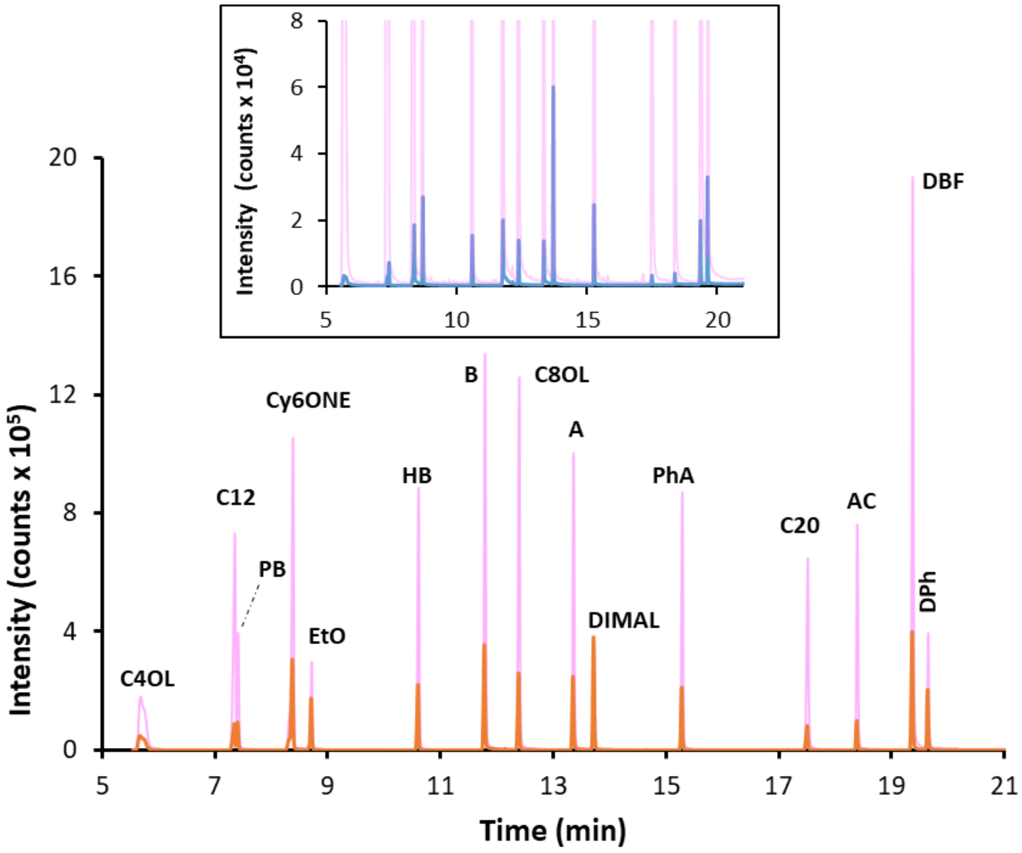

Figure S2. GC-combustion-MS chromatogram obtained for a mixture of three noncontaining (C12, C20 and AC) and twelve O-containing (C4OL, PB, Cy6ONE, EtO, HB, B, C8OL, A, DiMAL, PhA, DBF, DPh) compounds using ^{18}O-enriched oxygen (1% in He) as combustion gas. Compounds' abbreviations are given in the Experimental Section. Oxygen concentration ranged from 7.1 to 34.2 µg O g^{-1} with an average value of 17 µg O g^{-1}. Orange, blue, pink profiles correspond to 46, 44 and 48, respectively.

A)

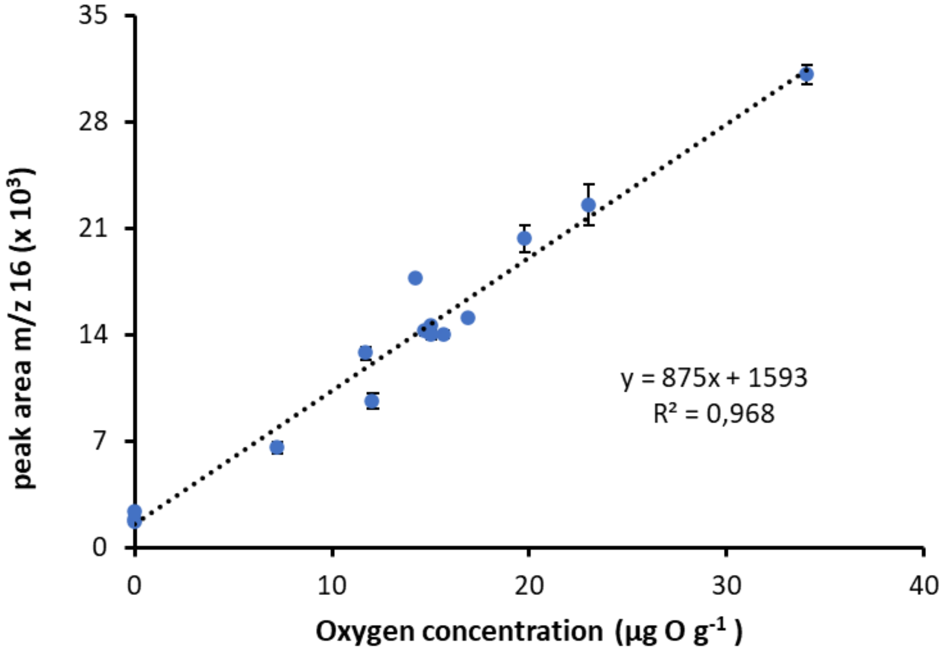

B)

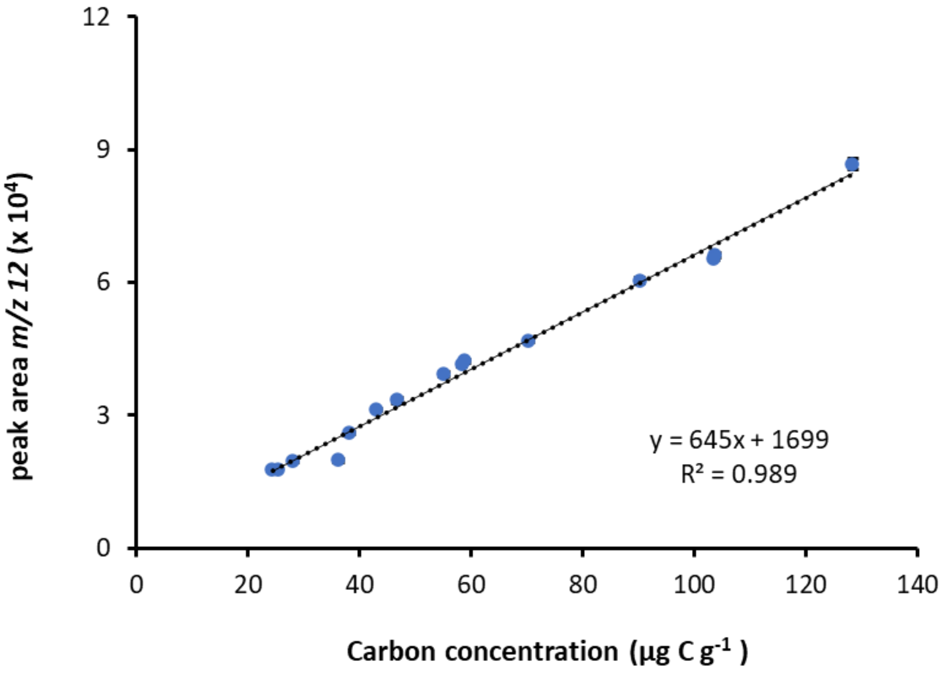

Figure S3. Oxygen (*m/z* 16, A) and Carbon (*m/z* 12, B) calibration curves obtained from the GC-combustion-MS chromatogram shown in Figure 2, which consists of a mixture of three noncontaining (C12, C20 and AC) and twelve O-containing (C4OL, PB, Cy6ONE, EtO, HB, B, C8OL, A, DiMAL, PhA, DBF, DPh) compounds using ^{18}O-enriched oxygen (1% in He) as combustion gas. Uncertainty bars correspond to 1 SD (n=3). Compounds' abbreviations and compound concentrations are given in the Experimental Section.

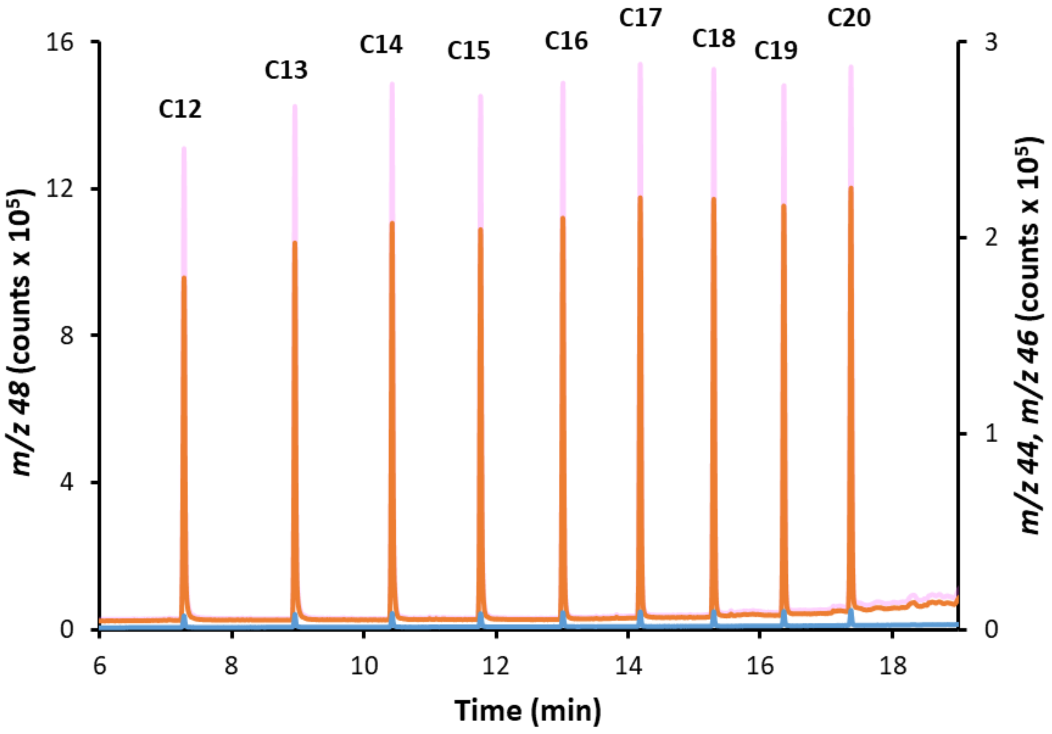

Figure S4. GC-combustion-MS chromatogram obtained for a mixture of eleven alkane compounds (C12-C20) using 18O-enriched oxygen (1% in He) as combustion gas. Blue, orange and pink profiles correspond to *m/z* 44, 46 and 48, respectively. Compounds' abbreviations and compound concentrations are given in the Experimental Section. Concentration of the compounds was 16 µg C g^{-1}.

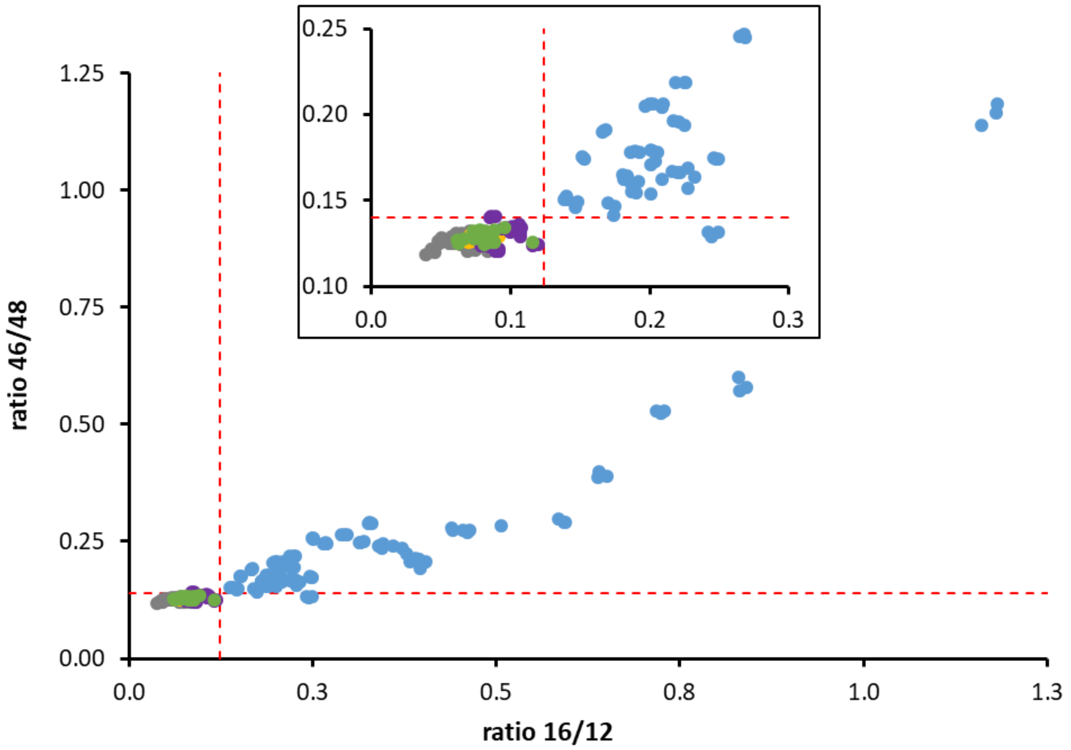

Figure S5. Plot of the ratios 16/12 vs 46/48 measured for each chromatographic peak detected in several mixtures of noncontaining and O-containing compounds and analyzed in triplicate on different working days. In total, 200 chromatographic peaks were processed, including 93 noncontaining (3 alkenes, 1 aromatic, 4 N-compounds, 4 S-compounds) and 107 O-containing peaks (13 O-compounds). Red dotted lines correspond to the limits of the 99% confidence interval for the non-containing O-compounds. Oxygen concentrations ranged from 4 to 37 µg O g^{-1}, with an average value of 14 µg O g^{-1}. See experimental section for details. Color code: alkanes (grey), aromatics (yellow), N-compounds (purple), S-compounds (green) and O-compounds (blue).

A)

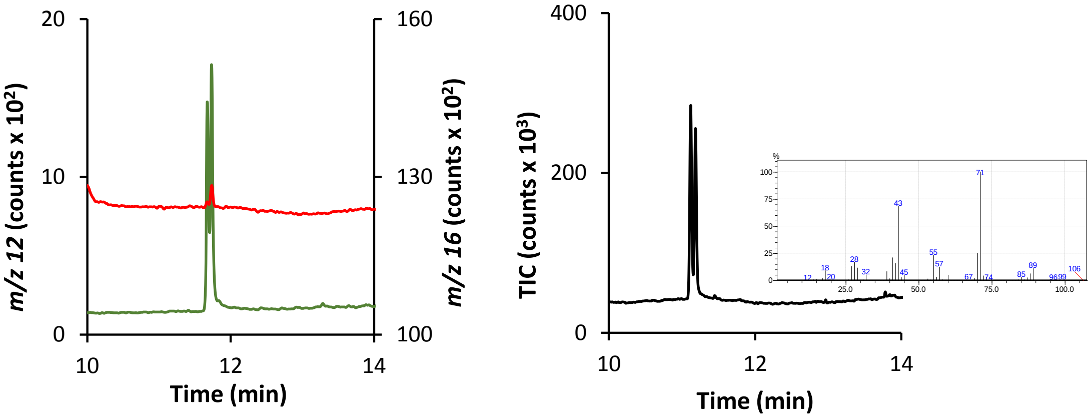
m/z 12 (counts x 10²)
m/z 16 (counts x 10²)
Time (min)
TIC (counts x 10³)
Time (min)

B)

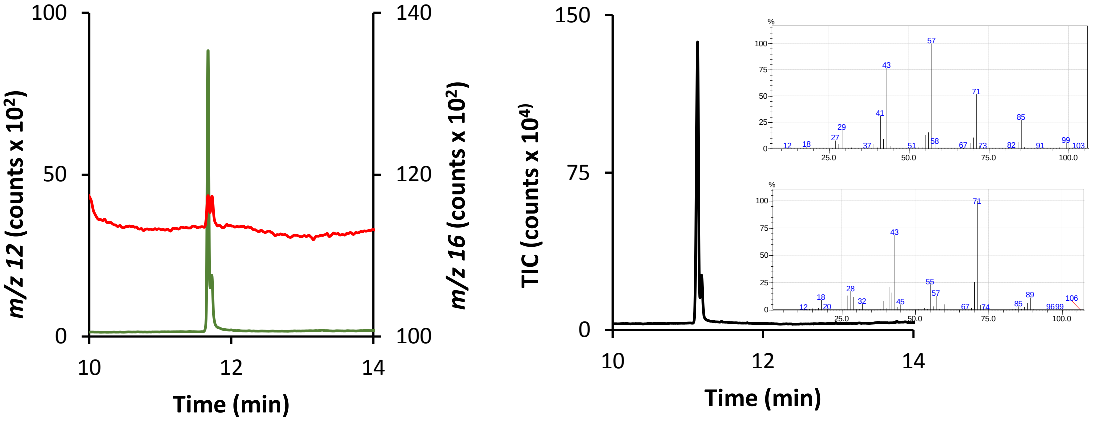
m/z 12 (counts x 10²)
m/z 16 (counts x 10²)
Time (min)
TIC (counts x 10⁴)
Time (min)

C)

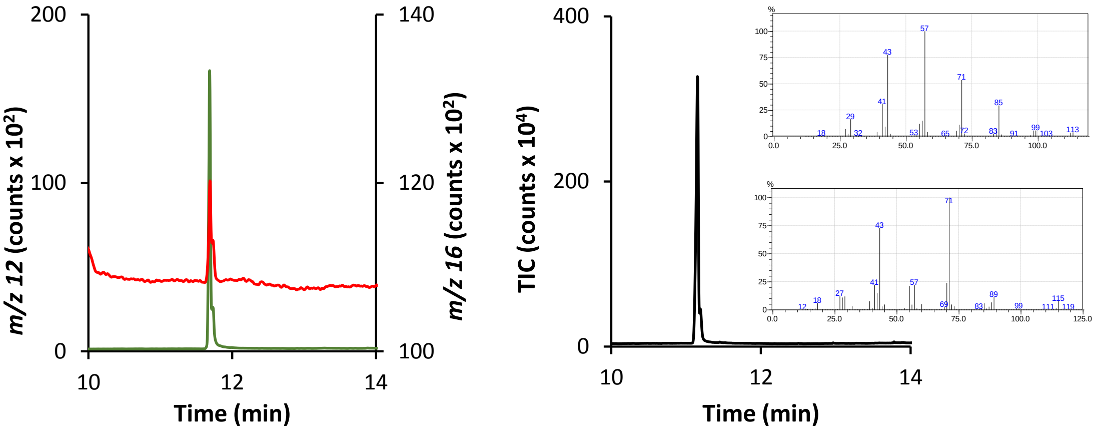
m/z 12 (counts x 10²)
m/z 16 (counts x 10²)
Time (min)
TIC (counts x 10⁴)
Time (min)

D)

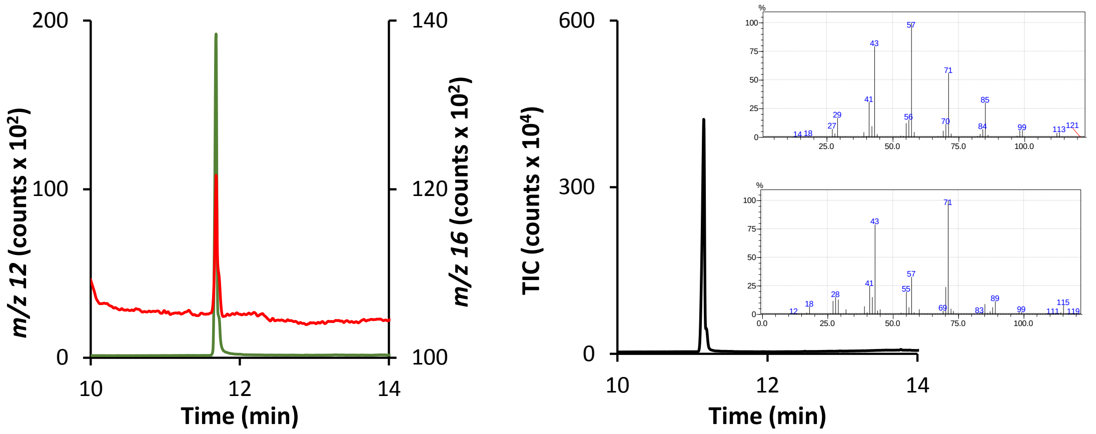

E)

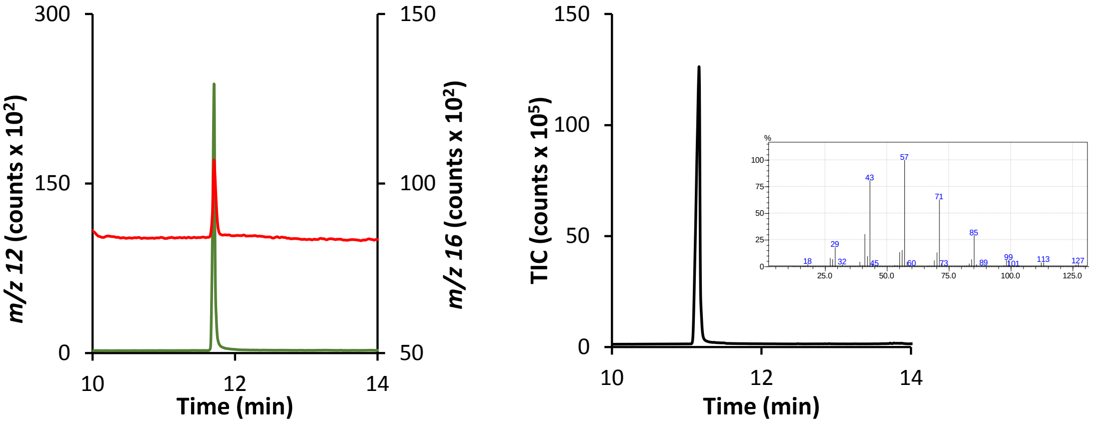

Figure S6. GC-combustion-MS (left panels) and GC-MS (right panels) chromatograms corresponding to low concentrate solutions (ca. 0.5-0.6 µg O g^{-1}) of an O-containing compound (2-Pentyl butyrate, retention time: 11.73 min) spiked with a noncontaining O-compound (C12, retention time: 11.67 min) at increasing concentration ratios: 1.1 (A) , 5.6 (B), 9.3 (C), 14.7 (D) and 18.4 (E). Left panels: orange and dark blue traces correspond to signals at *m/z* 12 and 16, respectively in the GC-combustion-MS chromatogram. Right panels: pale blue corresponds to the TIC signal in the GC-MS chromatograms and insets correspond to the corresponding MS spectra at the retention times of the individual peaks (A-D) and the unique-global peak (E).

A)

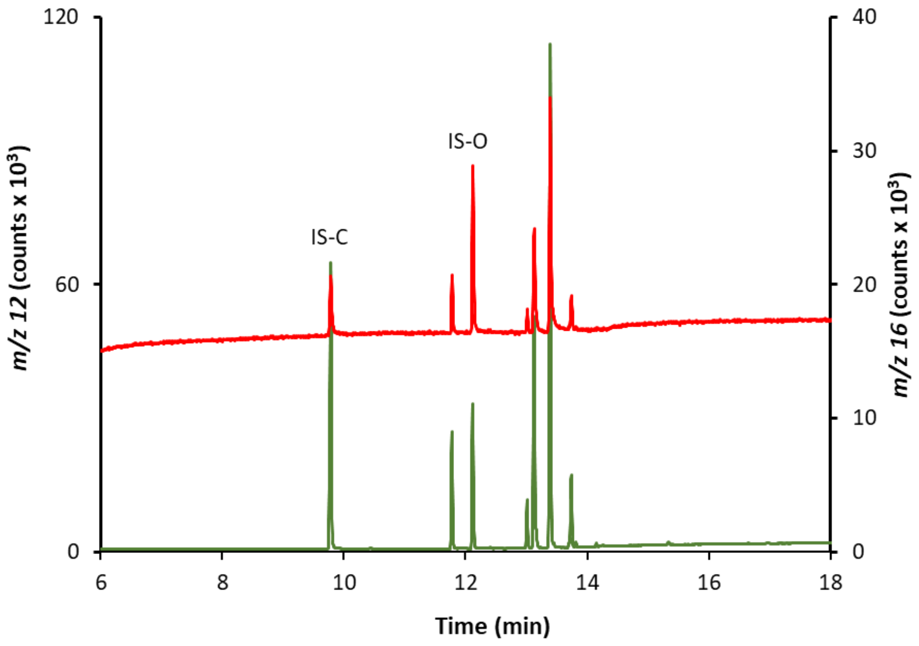

B)

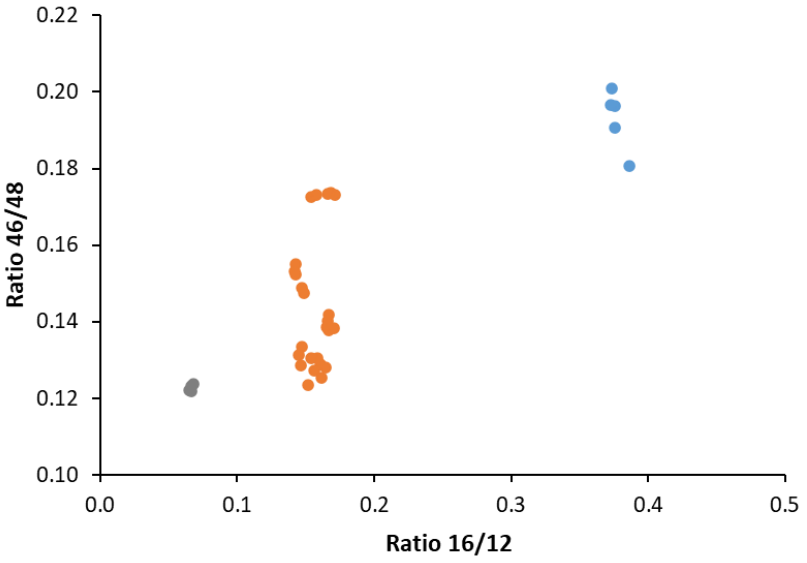

Figure S7. A) GC-combustion-MS chromatogram of an aliquot of the Biodiesel SRM 2772 previously spiked with Nonadecane and Dimethylphtalate as generic Internal Standards of C (IS-C) and O (IS-O). B) Plot of the ratios 16/12 vs 46/48 measured for each chromatographic peak detected in the quintuplicate analysis. Color code: IS-C (grey), IS-O (blue) and FAMEs (orange, O-compounds).

A)

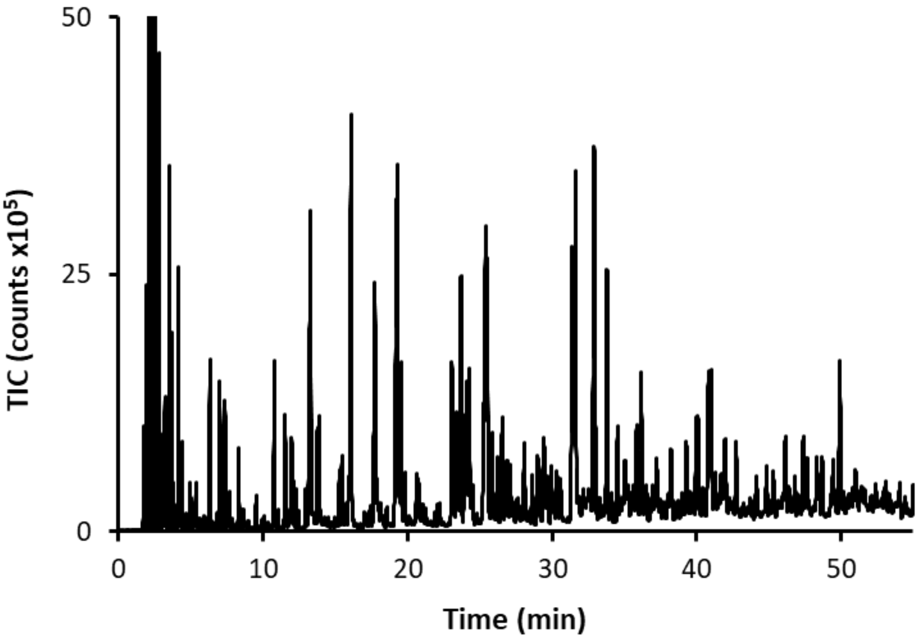

B)

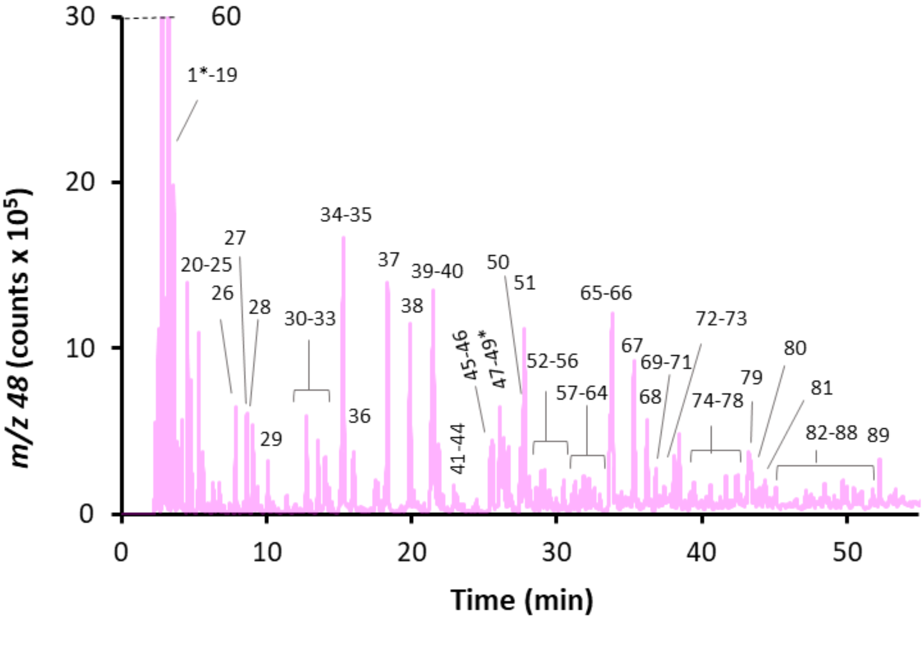

Figure S8. (A) GC-MS and (B) GC-combustion-MS at m/z 48 chromatograms of an aliquot of the effluent taken from the hydrotreatment process of a wood bio-oil (144.5 h of the catalyst life).
